# Transcriptomic and proteomic insight into the effects of a defined European mistletoe extract in Ewing sarcoma cells reveals cellular stress responses

**DOI:** 10.1186/s12906-017-1715-2

**Published:** 2017-04-28

**Authors:** M. Twardziok, D. Meierhofer, S. Börno, B. Timmermann, S. Jäger, Sengül Boral, A. Eggert, C. I. Delebinski, G. Seifert

**Affiliations:** 1 Department of Paediatric Oncology/Hematology, Otto Heubner Centre for Paediatric and Adolescent Medicine (OHC), Charité, Universitätsmedizin Berlin, Augustenburger Platz 1, 13353 Berlin, Germany; 2 Institute of Pharmacy, Department of Biology, Chemistry, Pharmacy, Freie Universität Berlin, Berlin, Germany; 3 Department of Paediatrics, Dr. von Haunersches Kinderspital, Klinikum der Universität München, Munich, Germany; 4 Max Planck Institute for Molecular Genetics, Berlin, Germany; 5 Birken AG, Niefern-Oeschelbronn, Germany; 6 Department of Pathology, Charité, Universitätsmedizin Berlin, Berlin, Germany

**Keywords:** Childhood cancer, Alternative medicine, Phytotherapy, ER stress, Oleanolic acid, Betulinic acid, MAPK

## Abstract

**Background:**

The hydrophobic triterpenes, oleanolic and betulinic acid as well as the hydrophilic mistletoe lectins and viscotoxins possess anticancer properties. They do all occur in combination in European mistletoe (*Viscum album* L.). Commercial *Viscum album* L. extracts are aqueous, excluding the insoluble triterpenes. We have previously shown that mistletoe lectins and triterpene acids are effective against Ewing sarcoma in vitro, ex vivo and in vivo.

**Methods:**

We recreated a total mistletoe effect (viscumTT) by combining an aqueous extract (viscum) and a triterpene extract (TT) solubilised with cyclodextrins and analysed the effects of viscumTT and the single extracts on TC-71 Ewing sarcoma cells in vitro by transcriptomic and proteomic profiling.

**Results:**

Treatment with the extracts strongly impacted Ewing sarcoma cell gene and protein expression. Apoptosis-associated and stress-activated genes were upregulated, proteasomal protein abundance enhanced and ribosomal and spliceosomal proteins downregulated. The mechanism of action of viscum, TT and viscumTT in TC-71 and MHH-ES-1 cells suggests the involvement of the unfolded protein response. While viscum and viscumTT extract treatment indicate response to oxidative stress and activation of stress-mediated MAPK signalling, TT extract treatment suggests the involvement of TLR signalling and autophagy.

**Conclusions:**

Since the combinatory extract viscumTT exerts highly effective pro-apoptotic effects on Ewing sarcoma cells in vitro, this phytopolychemotherapy could be a promising adjuvant therapeutic option for paediatric patients with Ewing sarcoma.

**Electronic supplementary material:**

The online version of this article (doi:10.1186/s12906-017-1715-2) contains supplementary material, which is available to authorized users.

## Background

Ewing sarcoma is the second most common form of bone sarcoma in children and adolescents [1], deriving from a mesenchymal stem cell or neuronal crest cell [2, 3]. Its pathogenesis results from a balanced translocation of the *EWS* gene creating fusion proteins which code for chimeric transcription factors promoting cell growth [4, 5]. Although 5-year survival in Ewing sarcoma patients is about 70%, the outcome for patients with metastatic disease or relapse drops to about 10–20% [1]. Resistance to the cytotoxic drugs used in conventional chemotherapy often occurs in persisting, recurrent or relapsed tumours, that may be avoided by specifically targeting pathogenetic mechanisms in Ewing sarcoma cells to kill cancer clones before resistance can be developed [6, 7]. Effective agents can also naturally occur in plant extracts, although their direct mechanisms of action may not be immediately clear.

The hemiparasite, *Viscum album* L. (European mistletoe), contains a large variety of different immunomodulatory and cytotoxic substances that can be highly effective against cancer cells. Active agents are primarily viscotoxins and mistletoe lectins I-III [8–10], but also include triterpenes and flavonoids [11–15]. Standardised aqueous mistletoe extracts are commercially available and popular in complementary cancer medicine. However, they contain only the hydrophilic mistletoe lectins and viscotoxins. Mistletoe lectins and also triterpene acids, such as betulinic acid or oleanolic acid and its derivatives, have been shown to inhibit cell growth and induce apoptosis in melanoma, breast cancer and leukaemia cells [16–18]. Despite the broad ranging anti-tumour effects of *Viscum album* L., there is little known about the signalling pathways affected during mistletoe-mediated apoptosis. Betulinic acid as well as oleanolic acid and its derivatives have been reported to activate stress-mediated MAPKs in gastric cancer, osteosarcoma, pancreatic cancer, breast adenocarcinoma, glioma and melanoma cells [19–23]. In leukaemia cells, mistletoe lectins were shown to activate MAPK8 [16, 24], and Korean mistletoe lectin was shown to activate TLR4 in dendritic cells [25]. But also AKT signalling has been implicated during mistletoe lectin or oleanolic acid treatment of gastric cancer, hepatocarcinoma, epidermoid cancer, colon carcinoma, ovarian cancer, prostate cancer, osteosarcoma and trophoblast cells, and oleanolic acid and its derivatives have been demonstrated to induce MTOR and NFKB1 signalling in prostate cancer, colon cancer and osteosarcoma cells [23, 26–34]. We have also previously demonstrated the therapeutic effect of recombining hydrophilic and hydrophobic mistletoe constituents in the viscumTT extract for Ewing sarcoma (Twardziok et al., 2016, manuscript accepted 07/2016) and acute leukaemia cells in vitro and in vivo cancer models [35, 36]. In Ewing sarcoma the mechanism leading to apoptosis involves the activation of caspases and the downregulation of the anti-apoptotic MCL1 and the IAP family members BIRC5 and XIAP. The aim of the present study was to analyse the impact of viscumTT and the single extracts on the transcriptome and proteome of Ewing sarcoma cells and to further illuminate the involved signalling pathways.

## Methods

### *Viscum album* L. extracts

Viscum and TT extracts were prepared from *Viscum album* L. harvested from apple trees (*malus*) as previously described [36] by the Birken AG (Niefern-Oeschelbronn, Germany), who kindly provided the lyophilized viscum and TT extracts for this study. Intact mistletoe lectin I (ML-I) was analysed by ELISA in viscum extract [37]. Oleanolic and betulinic acid were quantified, as a measure of extract activity, using GC-FID [18]. Lyophilized viscum extract was reconstituted in PBS (Gibco® Life Technologies, Darmstadt, Germany) to a final concentration of 2 μg/mL intact ML-I and <1 μg/mL viscotoxins. Lyophilized TT extract (containing cyclodextrins) was reconstituted in phosphate-buffered saline to a final concentration of 4000 μg/mL oleanolic and 350 μg/mL betulinic acid.

### Cell culture

Human Ewing sarcoma cell lines were obtained from the German Collection of Microorganisms and Cell Cultures (DSMZ, Braunschweig, Germany). The TC-71 and MHH-ES-1 cell lines were maintained in Iscove’s Modified Dulbecco’s Medium (Gibco Life Technologies) and RPMI 1640 base medium both supplemented with L-glutamine (Gibco Life Technologies), respectively. Base media were supplemented with 10% heat-inactivated FBS (Biochrom, Berlin, Germany), 100 U/mL penicillin and 100 μg/mL streptomycin (Biochrom). For assays, TC-71 cells were seeded into 6-well (2 × 10^5^) or 12-well (1 × 10^5^) microtiter plates or 25 cm^2^ (5 × 10^5^) or 75 cm^2^ (1.5 × 10^6^) cell culture flasks depending on experimental set-up. MHH-ES-1 cells were seeded into 6-well (4 × 10^5^) or 12-well (2 × 10^5^) microtiter plates. Cells were cultured 24 h to allow cell attachment, and treated 24 h with *Viscum album* L. extracts added to culture media. Viscum, TT and viscumTT concentrations were assessed by dose-effect-curves of apoptosis measurements as previously described [38].

### RNA isolation

TC-71 cells were incubated with increasing concentrations of the extracts for 24 h. RNA was isolated using the NucleoSpin® RNA Kit according to the manufacturer’s protocol (Macherey-Nagel, Düren, Germany) in five independent experiments. Purity and concentration was determined by OD260/280 on the NanoDrop™ 2000 spectrophotometer (Thermo Scientific, Waltham, MA, USA).

### mRNA sequencing and bioinformatics analysis

TC-71 cells were treated once with ~IC_50_ extract concentrations (viscum 2 ng/mL ML-I, TT 50 μg/mL oleanolic acid, viscumTT 1 ng/mL ML-I + 10 μg/mL oleanolic acid) for 24 h. After total RNA isolation from treated and control cells, Illumina TruSeq RNA sample preparation including a polyA selection step via oligo-dT beads was used to isolate mRNA and generate cDNA libraries. Samples were sequenced by paired-end mRNA sequencing on an Illumina HiSeq 2500 (50 bp reads, *n* = 1). Reads were mapped uniquely to human genome hg19 using CLC genomics software. Normalisation and identification of differentially expressed genes was performed using DE-Seq software [39] (Bioconductor open source software) to calculate fold-change relative to untreated control cells, false discovery rate (FDR) [40] and *p* value using the negative binomial distribution, with *p* ≤ 0.05 considered as significant. The heat map and Venn diagram illustrating differential gene expression as log_2_-fold change relative to untreated control cells was performed by using the R software package (http://www.r-project.org/). Gene enrichment and functional annotation analysis was performed with the top differentially expressed genes (*p* ≤ 0.01) using DAVID Bioinformatics Resources 6.7 NIAID/NIH (http://david.abcc.ncifcrf.gov/).

### cDNA synthesis and qPCR validation

Using 2 μg RNA from treated or control TC-71 cells, cDNA was synthesised using the High Capacity RNA-to-cDNA Kit according to the manufacturer’s protocol (Applied Biosystems, Waltham, MA, USA). To confirm mRNA sequencing results, gene expression was measured by real-time PCR on the StepOnePlus™ System in 96-well fast plates under standard conditions (10 min, 95 °C; 15 s, 95 °C and 60 s, 60 °C, 40×) using Power SYBR Green Master Mix including ROX as a passive reference (Applied Biosystems). The quantitative PCR reactions were 20 μl total volume containing 5 ng cDNA and 500 nM primers, which were predesigned and purchased from Integrated DNA Technologies (IDT, Leuven, Belgium): *DDIT3*: Hs.PT.58.14610020; *JUN*: Hs.PT.58.25094714.g; *MAP2K6*: Hs.PT.58.3312889; *GAPDH*: Hs.PT.39a.22214836. Primer efficiency was determined to be 90–100%. Expression was normalised using *GAPDH* expression. The relative expression of genes was calculated by ∆∆CT method: ΔΔCT = (CT(target,untreated) – CT(ref,untreated)) − (CT(target,treated) – CT(ref,treated)); fold-change =2^^(−∆∆CT)^ [41].

### Proteomic profiling and bioinformatics analysis

TC-71 cells were grown in 25 cm^2^ cell culture flasks in triplicate and treated with viscum, TT or viscumTT extracts at ~ IC_50_ concentrations (viscum 2 ng/mL ML-I, TT 50 μg/mL oleanolic acid, viscumTT 1 ng/mL ML-I + 10 μg/mL oleanolic acid) for 24 h. Cells were harvested and lysed under denaturing conditions in a buffer containing 6 M guanidinium chloride, 10 mM tris(2-carboxyethyl)phosphine, 40 mM chloroacetamide and 100 mM Tris pH 8.5. Lysates were sonicated and boiled at 95 °C for 5 min. Lysates were diluted 1:10 in 10% acetonitrile in 25 mM Tris, pH 8.5, and 2% of each total lysate volume was digested with 1 μg trypsin at 37 °C overnight. Peptides were acidified by adding formic acid to a final concentration of 1%, desalted using C18 StageTips (Thermo Scientific, Waltham, MA, USA) and lyophilised. Pellets were dissolved in 5% acetonitrile and 2% formic acid and one quarter of the digest was injected for nanoflow reverse-phase liquid chromatography (Dionex Ultimate 3000, Thermo Scientific) coupled online to a Thermo Scientific Q-Exactive Plus Orbitrap mass spectrometer (nanoLC-MS/MS). Briefly, the LC separation was performed using a PicoFrit analytical column (75 μm ID × 40 cm long, 15 μm Tip ID (New Objectives, Woburn, MA) packed in-house with 2.1 μm C18 resin (Reprosil-AQ Pur, Dr. Maisch, Ammerbuch-Entringen, Germany) under 50 C controlled temperature. Peptides were eluted using a nonlinear gradient from 2 to 40% solvent B in solvent A over 180 min at 266 nL/min flow rate. Solvent A was 0.1% formic acid and solvent B was 79.9% acetonitrile, 20% H_2_O, 0.1% formic acid). Nanoelectrospray was generated by applying 3 kV. A cycle of one full Fourier transformation scan mass spectrum (300–1700 m/z, resolution of 35,000 at m/z 200) was followed by 12 data-dependent MS/MS scans with normalised collision energy of 25 eV. A dynamic exclusion window of 30 s was used to avoid repeated sequencing of the same peptides. MS data were analysed by MaxQuant (v1.5.0.0) [42]. The algorithm MaxLFQ [43], which is integrated into MaxQuant, was used for label free quantification (LFQ). Peptides were searched against the human proteome UniProtKB database released in 11/2014 with 88,717 entries, released in 11/2014, using an FDR of ≤0.01 for proteins and peptides with a minimum length of seven amino acids. A maximum of two missed cleavages in the tryptic digest was allowed. Cysteine carbamidomethylation was set as a fixed modification, while N-terminal acetylation and methionine oxidation were set as variable modifications. Principal component analysis (PCA, Fig. 3b) and a heat map of the replicates (Fig. 3a) were carried out using the Perseus software (v1.5.1.6). Gene set enrichment analysis (GSEA, v2.1.0) [44] was applied to assess a priori defined protein sets with statistically significant expression differences between treated and control cells. We used GSEA standard settings, except that minimum size exclusion was set to five and KEGG v2.1.0 as well as reactome v5.0 was used as the gene set database. The String software tool (v10) was used to visualise protein-protein interaction networks of proteins up- or downregulated at least 2-fold [45]. Protein nodes that were not integrated into the protein-protein interaction network were removed.

### Western blotting

TC-71 and MHH-ES-1 cells were incubated with viscum, TT or viscumTT in increasing concentrations for 24 h. The cells were washed twice with phosphate-buffered saline and incubated in Lysis Buffer 17 (R&D systems, Minneapolis, MN) containing protease inhibitors (complete Protease Inhibitor Cocktail Tablets, Roche Diagnostics GmbH) to obtain cell lysates. Protein concentration was determined using Bradford solution (Bio-Rad, Munich, Germany). TC-71 and MHH-ES-1 cell lysates (30 μg protein/lane) were separated on SDS-PAGE, transferred to nitrocellulose membranes (Bio-Rad system) and blocked with 5% non-fat milk in 50 mM Tris-buffered saline containing 0.05% Tween-20 (TBST) for 1 h at room temperature. Blots were incubated overnight at 4 °C in TBST containing 5% BSA and primary antibody, washed thrice in TBST and incubated 1 h with HRP-conjugated secondary antibodies (anti-rabbit and anti-mouse, Bio-Rad) then visualized by ECL (Thermo Scientific) on a Molecular Imager ChemiDoc (Bio-Rad). Primary antibodies were directed against p-MAPK14 (Thr180/Tyr182, #9211 Cell Signaling Technology, Danvers, MA, USA), LC3B (#2775 Cell Signaling Technology), EIF2AK3 (#3192, Cell Signaling Technology), p-MAPK8 (sc-6254, Santa Cruz biotechnology, Dallas, TX, USA), HSPA5 (#G8918, Sigma-Aldrich) and ß-actin conjugated directly to peroxidase (#A3854, Sigma-Aldrich).

### Inhibitor treatment and apoptosis measurement

To measure the impact of TLR4 signalling, MAPK14 and MAPK8 activation or oxidative stress on apoptotic induction, TC-71 cells were pre-incubated with specific inhibitors for 1 h followed by an incubation with ~ IC_50_ extract concentrations for 24 h. Specific inhibitor treatment included 5–50 μM SB203580 (Cell Signaling Technology), 1 μM – 25 μM SP600125 (Sigma-Aldrich), 1–10 mM N-acetylcysteine (NAC, Sigma-Aldrich), 0.1–10 μg mL^−1^ LPS-RS (InvivoGen, San Diego, CA, USA). DMSO (Sigma-Aldrich) or PBS was added to extracts as solvent control, depending on the inhibitor diluent. After treatment, cells were washed twice with PBS, resuspended in 100 μl binding buffer and stained with APC-conjugated annexin V (BD Biosciences, Franklin Lakes, NJ, USA), according to the manufacturer’s protocol, then counterstained with 1 μl 1 mg/mL propidium iodide (Sigma Aldrich). Cells were analysed by flow cytometry (FACSCalibur, Becton Dickinson, Heidelberg, Germany), and the data were evaluated using FlowJo Software (TreeStar, Ashland, OR, USA).

### Statistical analyses

All qPCR experiments were performed in triplicate and experiments were repeated four times. Results are presented as mean values ± standard error of the mean (SEM). Inhibitor treatment and measurement of apoptosis was repeated thrice independently. The results are also presented as mean values ± SEM. Western blots were performed in three independent experiments. mRNA sequencing was performed in one experiment, while proteomic profiling was performed in triplicate. The cut-off for significantly deregulated pathways was set to *p* ≤ 0.01 for mRNAseq and *p* ≤ 0.05 and FDR ≤ 0.25 for proteomic profiling. Principal component analysis was performed to compare differences within the triplicates used for proteomic profiling. Protein-protein interaction networks of proteins up- or downregulated were regarded as significant by at least 2-fold change with a confidence level of 0.7 [45].

## Results

### ViscumTT alters the transcriptomic profile

**Fig. 1:**
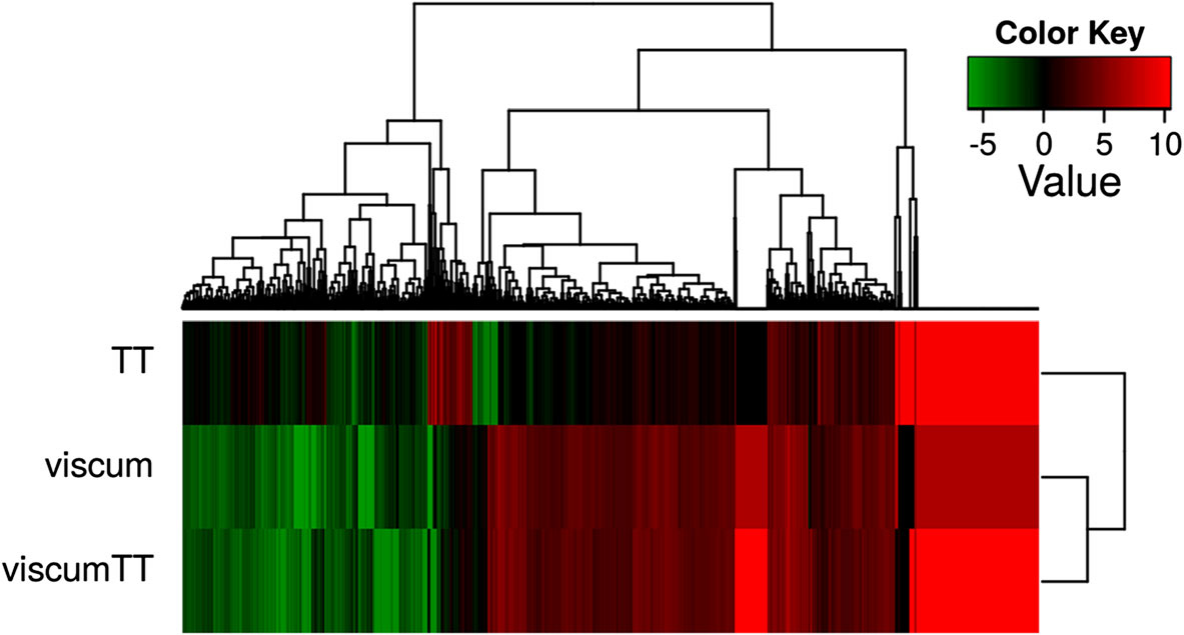
ViscumTT alters the transcriptomic profile. TC-71 cells were incubated for 24 h with viscumTT, viscum or TT in ~ IC50 concentrations followed by mRNA sequencing. Gene expression was calculated as the relative log_2_-fold change of untreated control cells in one experiment (*p* ≤ 0.05). Heat map displays genes upregulated relative to untreated control cells in *red* and downregulated genes in *green*

Firstly, to explore global changes asserted by mistletoe extracts in the Ewing sarcoma cell transcriptome, we treated TC-71 cells for 24 h with viscum, TT or viscumTT in ~ IC_50_ concentrations in reference to untreated control cells and performed one mRNA sequencing experiment. mRNA sequencing detected >62,400 transcripts belonging to >17,300 genes. Treatment with the extracts had a massive impact on the transcriptome of TC-71 cells, as shown in the heat map (hierarchical clustering) of log_2_-transformed fold-changes in gene expression (Fig. 1). Interestingly, treatment with viscumTT or viscum produced similar transcriptomic patterns of differentially expressed genes and resulted in a differential expression of >1000 genes, most of which were upregulated relative to the untreated control cells (Fig. 1). TT treatment displayed a different transcriptomic pattern and had a lesser impact on the TC-71 transcriptome, causing the differential expression of only 249 genes (Fig. 1). An overview of the differentially expressed genes is also given in a Venn diagram (Additional file 1: Figure S1).

**Fig. 2:**
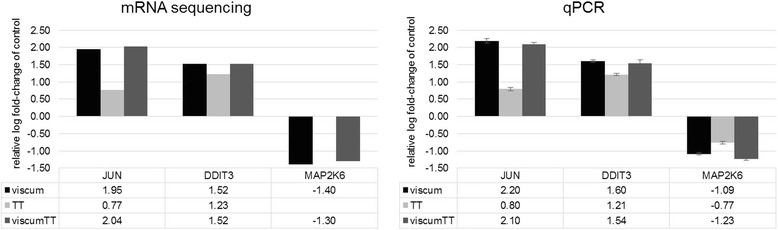
ViscumTT alters the expression of genes involved in MAPK signalling. TC-71 cells were incubated for 24 h with viscumTT, viscum or TT in ~ IC50 concentrations. The changes in *JUN*, *DDIT3* and *MAP2K6* expression detected using mRNA sequencing were validated using qPCR. Gene expression relative to untreated control cells was calculated from qPCR data using the ∆∆CT method. Bars represent the mean fold-change (log_10_ ± SEM, error bars) in gene expression in treatment groups from four independent qPCR experiments and one mRNA sequencing experiment

Several components of immune and cellular stress responses as well as cell survival and death pathways were within the genes deregulated by viscumTT, viscum or TT treatment. The top 40 genes (in terms of *p* ≤ 0.05) regulated by viscumTT, viscum or TT treatment of TC-71 cells are shown in Additional file 2: Tables S1, Additional file 3: Table S2 and Additional file 4: Table S3, respectively. For instance, *JUN*, *DDIT3* and *CXCL8* (formerly *IL8*) were upregulated and *MAP2K6* was downregulated by either viscumTT or viscum treatment within the top 40 deregulated genes, while *KLHDC7B*, *NUPR1* and *CYTH3* (formerly *GPR1*) were upregulated by TT treatment. *JUN* and *DDIT3* upregulation and *MAP2K6* downregulation were confirmed by qPCR (Fig. 2).

To look more deeply into pathways affected at the transcript level by mistletoe extract treatment, we preformed gene enrichment and functional annotation analysis using the DAVID bioinformatics tool. Since mRNA-sequencing experiment was performed only once, we chose a strict cut-off using exclusively genes with *p* ≤ 0.01 for differential expression changes from the control cells. ViscumTT and viscum treatment impacted functional annotation clusters for the positive regulation of cell death, response to reactive oxygen species/oxidative stress and MAPK signalling, while TT affected clusters for Toll-like receptor signalling, positive regulation of cell death and inflammatory response (Table 1). Taken together, the combined viscumTT extract as well as both single extracts altered expression of many genes, mostly upregulating them, in the TC-71 Ewing sarcoma cell line. ViscumTT or viscum treatment of TC-71 cells indicates a transcriptomic upregulation of cell stress and MAPK signalling genes, while TT appears to involve inflammatory response/TLR signalling, with both extract components transcriptionally exerting a pro-apoptotic effect on TC-71 Ewing sarcoma cells.

**Table 1:** Significantly regulated KEGG pathways in TC-71 cells treated 24 h with viscumTT, viscum or TT versus untreated control cells

Extract	Functional annotation	# Genes	*p*-Value	FDR
ViscumTT	MAPK signalling pathway	15	6.84 × 10^-6	6.70 × 10^-4
	Positive regulation of cell death	15	1.54 × 10^-4	1.54 × 10^-2
	Toll-like receptor signalling pathway	8	3.24 × 10^-4	1.58 × 10^-2
	Oxidative stress response	6	1.06 × 10^-3	1.95 × 10^-2
	Apoptosis	19	4.41 × 10^-5	2.84 × 10^-2
Viscum	Response to reactive oxygen species	8	2.59 × 10^-6	1.35 × 10^-3
	Response to organic substance	19	2.17 × 10^-5	7.49 × 10^-3
	Positive regulation of cell death	12	8.81 × 10^-4	7.34 × 10^-2
	MAPK signalling pathway	10	1.75 × 10^-3	1.43 × 10^-1
TT	Toll-like receptor signalling pathway	3	4.86 × 10^-2	7.64 × 10^-1
	Positive regulation of cell death	5	4.52 × 10^-2	9.95 × 10^-1
	Inflammatory response	5	1.79 × 10^-2	1.00 × 10^+0

*p ≤ 0.05 and FDR ≤ 0.1 regarded as significant

### ViscumTT alters the proteomic profile

**Fig. 3:**
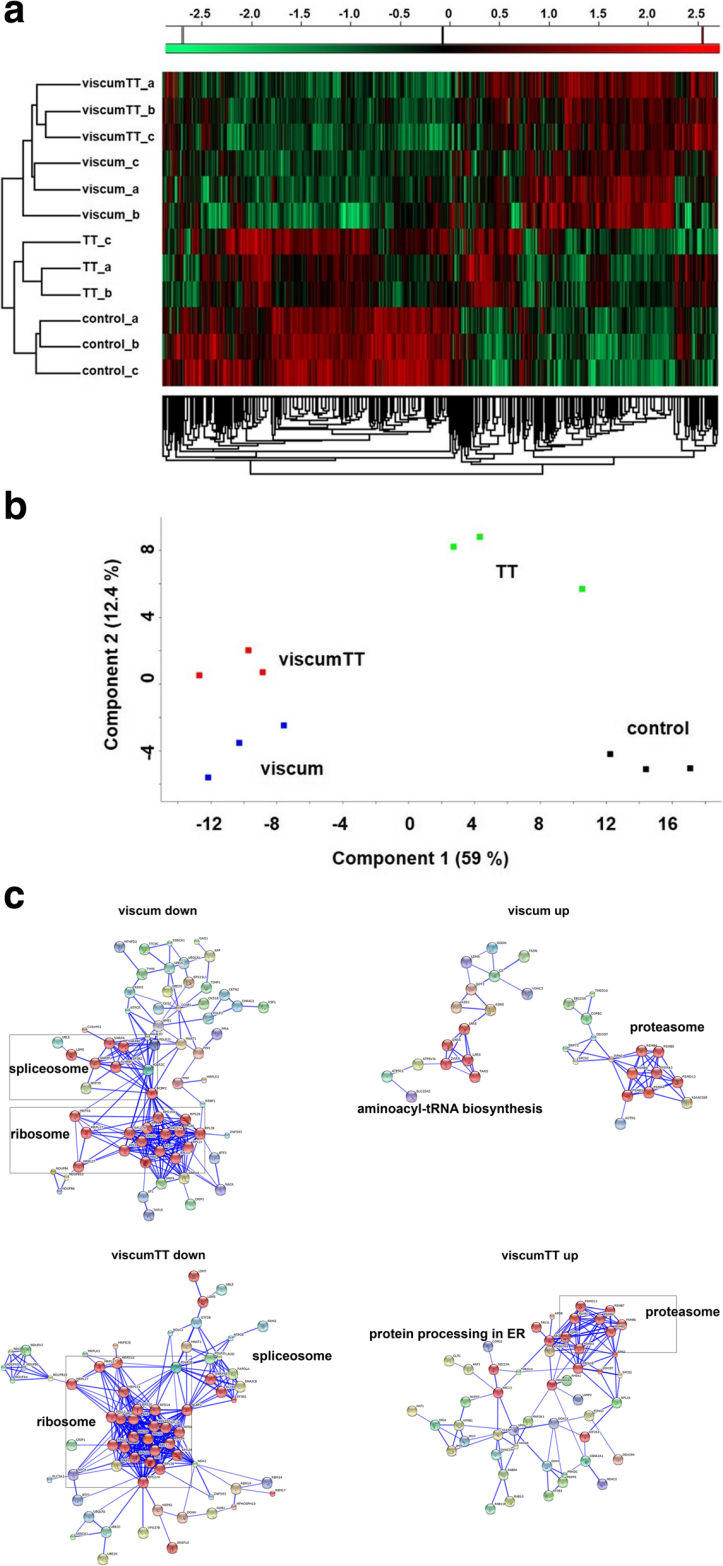
ViscumTT alters the proteomic profile. TC-71 cells were treated as biological triplicates with viscumTT or each single extract (~ IC50) for 24 h and analysed by LC − MS/MS. **a** To display differential protein expression in sample cohorts, a heat map was created by Perseus software using the z-score (standard score) of log2-transformed ion intensities. **b** Principal component analysis of the replicates was performed to compare the effects of *Viscum album* L. extract treatment. **c** String network analysis of the averaged replicates was performed to detect protein-protein interaction networks. Proteins indicated in *red* belong to the specified KEGG pathway

We next extended our investigations of mistletoe extract component effects to changes exerted on the TC-71 Ewing sarcoma cell proteome using LC − MS/MS technology. We examined the same temporal and dose response window (24 h and ~ IC_50_ concentrations) as for transcriptomic changes. To get more reliable results, TC-71 cells were treated in three independent experiments with viscumTT, viscum or TT in reference to untreated control cells followed by subsequent analysis by LC − MS/MS. More than 3 × 10^4^ MS spectra were identified in each sample, which could be mapped to 3554 proteins in total. The ion intensities were log_2_-transformed and normalised using the z-score by Perseus software and then plotted as a heat map (hierarchical clustering) to display the relative protein expression levels in the different TC-71 cell treatments and untreated control cells. Similar to the transcriptomic results, the heat map displayed an impressive alteration of the proteome after treatment with viscumTT or viscum compared to control cells, whereas TT treatment showed fewer changes (Fig. 3a). Principal component analysis verified good compliance between the experimental replicates (Fig. 3b). Therefore, LFQ ion intensities from the experimental replicates were averaged for pathway analyses, only valid values were used. The top 40 differentially expressed proteins (in terms of *p* ≤ 0.05) are presented in Additional file 5: Tables S4, Additional file 6: Table S5 and Additional file 7: Table S6. To look more deeply into pathways affected at the protein level by mistletoe extract treatment, we preformed gene set enrichment analysis using the GSEA bioinformatics tool. Downregulated proteins after treatment with viscumTT, viscum or TT related to ribosome/translation and transcription (Table 2). Protein-protein interaction network analyses of proteins downregulated by at least 2-fold revealed for viscumTT or viscum treatment proteins involved in the ribosome and spliceosome, whereas TT treatment was not linked with any functional protein networks (Fig. 3c). Gene set enrichment analysis also revealed that viscumTT, viscum or TT treatment upregulated proteins linked with aminoacyl-tRNA biosynthesis and the proteasome. Additionally, treatment with viscumTT or viscum also upregulated proteins associated with the regulation of apoptosis, protein folding, PERK-regulated gene expression, MAP kinase activation downstream of the toll-like receptor and the immune system (Table 3). Protein-protein interaction network analyses of proteins upregulated at least 2-fold by viscumTT treatment implicated both the protein processing network in the endoplasmic reticulum (ER) and the proteasome, while viscum treatment implicated aminoacyl-tRNA synthesis and the proteasome (Fig. 3c). Our data indicate that treatment with viscum, TT or the combined viscumTT extracts results in a reduction of proteins related to the ribosome and spliceosome, while increasing proteins associated with the proteasome. Treatment with either viscumTT or viscum also triggers protein upregulation associated with apoptosis, protein folding and MAPK signalling.

**Table 2:** Significantly regulated KEGG and Reactome pathways in TC-71 cells treated 24 h with viscumTT, viscum or TT versus untreated control cells

Extract	Pathway	# Proteins	*p*-Value	q-Value
	>Downregulated			
Viscum	Ribosome	75	0.00	0.00
	Transcription	57	0.00	9.64 × 10^+3
	Translation	122	7.95 × 10^-3	1.80 × 10^-1
TT	Ribosome	76	0.00	8.58 × 10^-3
	Transcription	59	0.00	3.18 × 10^-2
	Translation	123	7.11 × 10^-3	2.10 × 10^-1
ViscumTT	Ribosome	76	0.00	0.00
	Transcription	59	0.00	0.00
	Translation	124	5.95 × 10^-3	1.20 × 10^-1
	>Upregulated			
Viscum	Aminoacyl-tRNA biosynthesis	22	0.00	0.00
	Proteasome	35	2.96 × 10^-3	9.81 × 10^-2
	Protein folding	28	2.98 × 10^-3	3.89 × 10^-2
	PERK regulated gene expression	10	1.27 × 10^-2	6.36 × 10^-2
	MAP kinase activation in TLR cascade	8	5.64 × 10^-2	1.15 × 10^-1
	Immune system	175	0.00	1.51 × 10^-1
TT	Aminoacyl-tRNA biosynthesis	22	0.00	0.00
	Proteasome	35	1.08 × 10^-2	3.48 × 10^-2
ViscumTT	Aminoacyl-tRNA biosynthesis	22	0.00	0.00
	Proteasome	35	0.00	5.72 × 10^-2
	Regulation of apoptosis	38	0.00	3.10 × 10^-2
	PERK regulated gene expression	10	1.80 × 10^-2	6.42 × 10^-2
	Immune system	175	3.00 × 10^-3	7.35 × 10^-2
	Protein folding	28	1.42 × 10^-2	7.91 × 10^-2
	MAP kinase activation in TLR cascade	8	3.90 × 10^-2	7.95 × 10^-2
	Apoptosis	69	2.85 × 10^-2	1.59 × 10^-1

*p ≤ 0.05 and q ≤ 0.25

**Table 3:** Tested inhibitors

Inhibitor	Concentration	Tolerance
SB203580	5–50 μM	10 μM
SP600125	10 nM - 25 μM	5 μM
LPS-RS	0.1–10 μg/mL	0.1 μg/mL
NAC	1–10 mM	10 mM

### ViscumTT induces cellular stress responses

**Fig. 4:**
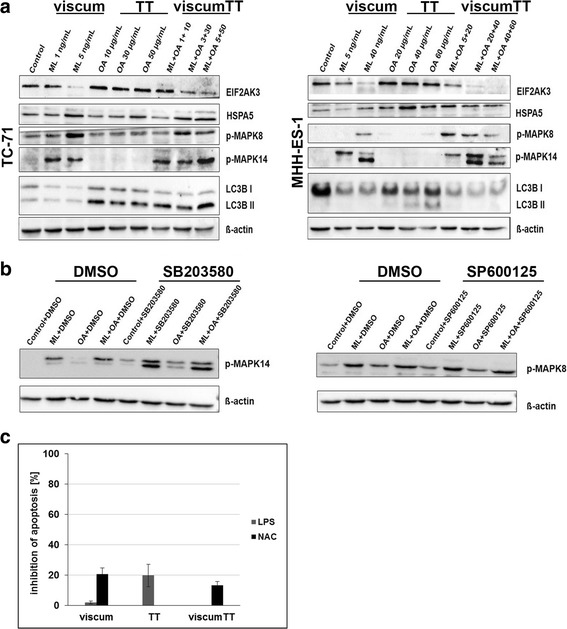
ViscumTT induces cellular stress responses. **a** TC-71 and MHH-ES-1 cells were treated with increasing concentrations of viscumTT (ML-I 1–40 ng/mL + OA 10–60 μg/mL), viscum (ML-I 1–40 ng/mL) or TT (10–60 μg/mL) for 24 h. Activation of stress-mediated MAPK signalling (p-MAPK8, p-MAPK14), cellular stress/unfolded protein response (EIF2AK3, HSPA5) and autophagy (LC3BI/II) were assessed using western blotting. β-actin was used as loading control, and images shown are representative for three independent experiments. Mistletoe lectin (ML) and oleanolic acid (OA) concentrations were used as a measure of viscum and TT active agent extract concentration, respectively. **b** TC-71 cells were treated with viscumTT, viscum or TT (~ IC50 concentration) for 24 h in the presence of DMSO (inhibitor solvent control), 10 μM SB203580 or 5 μM SP600125 to inhibit MAPK14 or MAPK8 activation, respectively. Inhibition of MAPK activation was not detected within the used inhibitor concentrations in whole-cell extracts using western blotting. β-actin was used as loading control, and images are representative for results from three independent experiments. **c** TC-71 cells were treated with viscumTT, viscum or TT (~ IC50 concentration) for 24 h in the absence or presence of the TLR4 inhibitor, LPS-RS (0.1 μg/mL), or the antioxidant, N-acetylcysteine (NAC, 5 mM). Apoptosis was flow cytometrically assessed following annexin V/propidium iodide staining. Bars show the percentage of apoptosis inhibition is shown in bars (±SEM) from three independent experiments

We next aimed to analyse the activation of cellular stress responses upon mistletoe extract treatment using western blotting to validate corresponding hints from transcriptomic and proteomic results. Activation of stress-mediated MAPK signalling by viscumTT and viscum was confirmed by increased phosphorylation of MAPK8 (formerly JNK) and MAPK14 (formerly p38/MAPK) in TC-71 and MHH-ES-1 cells 24 h after treatment with the extracts (Fig. 4a). All mistletoe extracts upregulated expression of the HSPA5 ER-chaperone protein, indicating response to cellular stress (e.g. oxidative stress or ER stress) by activation of the unfolded protein response (Fig. 4a). ViscumTT and viscum also reduced levels of the EIF2AK3 ER-stress sensor protein, whereas TT slightly increased EIF2AK3 levels (Fig. 4a). Since ER stress is linked to autophagy [46, 47], we next checked LC3B expression, whose conversion from LC3B-I to LC3B-II is a marker for autophagy. ViscumTT and TT increased LC3B-II levels in TC-71 cells, whereas only TT increased LC3B-II levels in MHH-ES-1 cells (Fig. 4a).

In order to further analyse the role of stress-mediated MAPK or TLR signalling and oxidative stress in mistletoe-mediated apoptosis of Ewing sarcoma cells, TC-71 cells were treated with viscumTT, viscum or TT for 24 h in the absence or presence of the SB203580 MAPK14 inhibitor, the SP600125 MAPK8 inhibitor, the LPS-RS TLR4 inhibitor or the antioxidant NAC. 5 μM MAPK8 and 10 μM MAPK14 inhibitor treatment was not able to prevent apoptotic induction by mistletoe extracts. Western blot analyses of TC-71 cell lysates after inhibitor treatment revealed no reduction in MAPK8 phosphorylation and, unexpectedly, enhanced MAPK14 phosphorylation (Fig. 4b). Higher MAPK8 and MAPK14 inhibitor concentrations (up to 50 μM SB203580 and 25 μM SP600125), as well as higher concentrations of the other used inhibitors (Table 3), all increased apoptosis in TC-71 cells. Pre-treatment with the antioxidant NAC (5 mM), however, reduced apoptosis by 21% after viscum treatment and ~13% after viscumTT treatment (Fig. 4c). LPS-RS (0.1 μg/mL), which binds TLR4 but prevents receptor activation, reduced TT-mediated apoptosis by ~20% (Fig. 4c). Taken together, these data indicate that viscumTT, viscum and TT activate the unfolded protein response. TT extract treatment suggests the activation of TLR4 signalling and autophagy, while viscumTT and viscum indicate the induction of oxidative stress and stress-mediated MAPK signalling. Since mistletoe extracts appear to trigger multiple pathways sensitizing Ewing cells to apoptosis, inhibiting single pathways remained insufficient to prevent apoptosis in combination with extract treatment.

## Discussion & conclusions

ViscumTT reconstitutes the aqueous lectins and viscotoxins as well as the hydrophobic (with cyclodextrins solubilised) triterpene acids of mistletoe into a defined total extract, combining all components predicted to have cytotoxic properties. ViscumTT extract treatment significantly altered both the transcriptome and proteome of TC-71 cells affecting cellular stress response pathways related to cell death. Upregulation of cellular stress associated proteins was also confirmed in MHH-ES-1 cells. While viscum treatment alone displayed similar effects on the transcriptome and proteome of TC-71 cells, the TT extract alone showed different and less alterations. On the one hand, it is possible that viscumTT and TT treatment displayed different significantly deregulated genes and proteins due to the very different olanolic acid concentrations used in TT and the synergistically apoptosis-inducing viscumTT extract to reach IC50 (TT 50 μg/mL oleanolic acid, viscumTT 10 μg/mL oleanolic acid), while the difference between ML-1 concentration in viscum (2 ng/mL ML-1) and viscumTT (1 ng/mL ML-1) extract treatment were more alike. On the other hand, it is conceivable that TT-activated pathways are different from those activated by viscum or viscumTT. We have previously demonstrated that viscum and even more, viscumTT effectively induced apoptosis in Ewing sarcoma cells in vitro and ex vivo accompanied by a loss of mitochondria membrane potential and activation of CASP-8, −9 and −3, while TT alone showed only moderate apoptosis induction without a significant loss of mitochondria membrane potential and activation of CASP-8, −9 and −3. However, TT potentiates the effect of viscum resulting in a synergistic apoptosis-induction by viscumTT (Twardziok et al. 2016, manuscript accepted).

We show here that viscum, TT and total mistletoe viscumTT extract affect different transcriptomic changes in Ewing sarcoma TC-71 cells. Both, viscumTT and viscum, upregulated *JUN* and other genes involved in cell death, MAPK signalling and oxidative stress. TT upregulated genes involved in TLR signalling and cell death as well as genes involved in inflammatory response. Although our results are preliminary due to a limited validity of one single mRNA-sequencing experiment for each extract treatment, others reported similar findings after mistletoe extract treatment. For instance, Yang et al. reported that recombinant mistletoe lectin differentially regulated several genes in the MAPK signalling cascade in hepatocellular carcinoma cells [26]. Furthermore, a similar transcriptomic profile involving apoptosis and MAPK signalling pathway was shown in breast cancer cells using DNA microarray chips after treatment with an commercial aqueous mistletoe extract [48]. Interestingly, they showed that aqueous mistletoe extracts from oak and apple tree hosts more strongly upregulate immune defence and stress response in breast cancer cells, whereas aqueous extracts from the host tree white fir affect cell-cell adhesion and cytoskeleton pathways. In line with this, our extracts from the apple tree as host evoked cellular stress and immune defence responses.

We further show that viscumTT, viscum and TT affect different proteomic changes in TC-71 cells. Treatment with the extracts resulted in the downregulation of proteins involved in translation and transcription/spliceosome and an upregulation of proteins involved in aminoacyl-tRNA biosynthesis and the proteasome. ViscumTT and viscum contain mistletoe lectins, which are classified as ribosome-inactivating proteins type-II provoking a breakdown of translation [49–51] leading to proteasomal degradation. Ribosome-inactivating proteins type-II have n-glycosidase activity removing single adenines from rRNA and they were originally thought to act exclusively on ribosomes, but there is evidence growing that they are also able to inactivate non-ribosomal nucleic acid substrates [52, 53]. Therefore, it is conceivable that they also affect the spliceosome, which consists of snRNAs and protein complexes. ViscumTT and viscum treated TC-71 cells also displayed an upregulation of proteins involved in protein folding, regulation of apoptosis, immune system and MAPK activation correlating with the transcriptomic results and suggesting that viscumTT and viscum have an impact on MAPK signalling and protein folding. Taken together, our transcriptomic and proteomic data showed that the triterpene extract TT had a lower influence on the transcriptome and proteome of TC-71 cells than viscum and viscumTT, which displayed similar profiles. The mistletoe extracts affect transcription, translation and the proteasome and they affect cellular stress and inflammatory responses. However, the similar proteomic and transcriptomic profiles of viscumTT and viscum treated TC-71 cells suggest that the synergistic effect of viscumTT is not created on transcriptional or translational level.

Our deeper assessment of the affected signalling pathways upon mistletoe treatment revealed the activation (phosphorylation) of stress-mediated MAP kinases MAPK8 and MAPK14 by viscumTT and viscum in TC-71 and MHH-ES-1 cells. In agreement with our results, others have shown the activation of MAPK8 signalling by an aqueous European and Korean mistletoe extract in leukaemia and hepatocarcinoma cells [16, 54, 55]. Other ribosome-inactivating proteins as Ricin or Shiga toxins also activate MAPK8 and MAPK14 in human monocytes and macrophages [56–58]. Contrary to our results, the triterpenes oleanolic and betulinic acid were also shown to activate MAPK8 and MAPK14 in melanoma, pancreatic cancer and osteosarcoma cells and hypertrophic scar fibroblasts [19, 21, 59]. Since the activation of MAPK8 and MAPK14 is stress-mediated resulting in apoptosis and because our pathway analyses displayed response to oxidative stress and TLR signalling, we next investigated the impact of MAPK8, MAPK14 and TLR4 inhibitors and the anti-oxidant NAC on apoptosis induction in TC-71 cells upon treatment with the extracts. TT-mediated apoptosis was reduced when cells were pre-treated with the TLR4 antagonist LPS-RS suggesting the involvement of TLR4 signalling by TT treatment. TLR4 signalling has been described in mouse splenocytes after treatment with an oleanolic acid derivative [60]. With regard to oxidative stress, NAC pre-treatment slightly reduced apoptosis by viscumTT and viscum indicating that oxidative stress is involved in viscumTT- and viscum-mediated apoptosis. In line with our results, the induction of oxidative stress by aqueous mistletoe extracts was shown in leukaemia and hepatocarcinoma cells [16, 55]. However, an oleanolic acid derivative was also reported to induce oxidative stress in ovarian cancer cells [61]. Notably, the applied MAPK14 and MAPK8 inhibitors (up to 10 μM SB203580, 5 μM SP600125) were not able to inhibit the activation of the kinases and to prevent the induction of apoptosis by viscumTT or viscum. Higher concentrations of the inhibitors resulted in a significant loss of cell viability demonstrating the cytotoxic potential of the inhibitors in TC-71 cells (data not shown). Others have used superior concentrations (up to 30 μM SB203580 or SP600125) to achieve a block in mistletoe mediated apoptosis induction without a loss of cell viability in leukaemia and hepatocarcinoma cells suggesting that higher concentrations might be necessary to effectively block apoptosis [16, 55, 62]. However, it is likely that the MAPK pathway is not the major pathway engaged by the extracts since viscumTT and viscum displayed an impressive impact on the transcriptome and proteome of TC-71 cells suggesting that the reasons for the apoptosis induction by the extracts are manifold. In summary, our deeper assessment of the activated signalling pathways indicates the involvement of TLR4 signalling for the TT extract and the activation of the stress-mediated MAPK signalling and oxidative stress for viscum and viscumTT. However, a deeper investigation of the activated pathways is needed.

Since there is a crosstalk between oxidative stress and the unfolded protein response and ER stress is also linked to MAPK8 activation [63–66], we analysed the protein expression of the ER chaperone protein HSPA5 [67] and the ER stress sensor protein EIF2AK3 [68, 69] in TC-71 and MHH-ES-1 Ewing sarcoma cells. We detected an upregulation of HSPA5 by viscumTT and both single extracts in both cell lines suggesting response to oxidative or ER stress by activation of the unfolded protein response. However, our results are preliminary and need further specific analyses for validation. Interestingly, the induction of HSPA5 and DDIT3 (formerly CHOP) leading to ER stress was already reported for a commercial aqueous extract in leukaemia cells [16]. We also detected a transcriptional an upregulation of *DDIT3* after treatment with viscumTT or both single extracts. Other ribosome-inactivating proteins like Ricin and Shiga toxins were also shown to activate the unfolded protein response in human breast and colon cancer cell lines [70] and a rat renal tubular epithelial cell line [71]. Furthermore, the naturally occurring triterpenoid celastrol induced the unfolded protein response in head and neck cancer cell lines [72]. Notably, viscumTT and viscum treatment concurrently displayed a decrease of EIF2AK3, whereas TT treatment showed a slight upregulation matching ER stress. Others demonstrated the induction of apoptosis by ER stress in HeLa cells after treatment with an oleanolic acid derivative [73]. Proteomic investigation of betulinic acid-induced apoptosis in HeLa cells also displayed the induction of ER stress [74]. Furthermore, an oleanolic acid derivative was shown to trigger ER stress leading to MAPK8-dependent apoptosis [75]. Because ER stress is connected to autophagy [46, 47, 76, 77], we also analysed the expression of the autophagy marker LC3B. The triterpene extract TT increased LC3B-II expression in both cell lines indicating enhanced autophagic activity. Consistent with our results, the triterpenes ursolic, betulinic or oleanolic acid and its derivatives were reported to induce autophagy in glioblastoma, breast cancer, prostate cancer, myeloma, KRAS transfected MCF10A breast epithelial cells as well as in Human Embryonic Kidney 293 cells, human hepatic cells, immortalized breast epithelial, gastric mucosal and primary bladder epithelial cells [78–84]. Others, by contrast, reported autophagy induction by Korean mistletoe lectin in placenta-derived mesenchymal stem cells [85]. In summary, viscumTT and the single extracts viscum and TT influence protein folding and indicate the involvement of the unfolded protein response and autophagy (TT).

In conclusion, we provide a first deep insight into the effects of single and combined mistletoe extracts in Ewing sarcoma cells in vitro. ViscumTT and both single extracts induce diverse cellular stress responses and influence protein folding. While viscumTT and viscum suggest the activation of the stress-mediated MAPK signalling and induction of oxidative stress, the TT extract indicates the induction of autophagy and TLR4 signalling. As viscumTT and viscum appear to activate the same signalling pathways differing from TT-activated pathways, the synergistic effect of viscumTT demonstrated in our previous work cannot be explained so far. However, the results suggest that the synergistic effect of viscumTT is created by other mechanisms. Nevertheless, viscumTT, which combines the anticancer effects of hydrophilic and hydrophobic mistletoe compounds, may represent a promising adjuvant phytopolychemotherapeutic therapy option for Ewing sarcoma patients.

## Additional files

Additional file 1: Figure S1. Differentially expressed genes after treatment with viscum, TT or viscumTT in TC-71 cells. TC-71 cells were incubated for 24 h with viscumTT, viscum or TT in ~ IC50 concentrations in reference to untreated control cells followed by one mRNA sequencing experiment. Normalisation and identification of differentially expressed genes was performed using DE-Seq software (Bioconductor open source software) to calculate fold-change relative to untreated control cells, false discovery rate and *p* value using the negative binomial distribution, with *p* ≤ 0.05 considered as significant. Venn diagram displays uniquely and commonly deregulated genes by the extracts.

Additional file 2: Table S1. The 40 most significantly regulated genes by viscumTT treatment (24 h) in TC-71 cells as fold-change relative to untreated control cells. (DOCX 14 kb)

Additional file 3: Table S2. The 40 most significantly regulated genes by viscum treatment (24 h) in TC-71 cells as fold-change relative to untreated control cells. (DOC 78 kb)

Additional file 4: Table S3. The 40 most significantly regulated genes by TT treatment (24 h) in TC-71 cells as fold-change relative to untreated control cells. (DOC 77 kb)

Additional file 5: Table S4. The 40 most significantly regulated proteins by viscumTT treatment (24 h) in TC-71 cells as fold-change relative to untreated control cells (DOC 79 kb)

Additional file 6: Table S5. The 40 most significantly regulated proteins by viscum treatment (24 h) in TC-71 cells as fold-change relative to untreated control cells. (DOC 79 kb)

Additional file 7: Table S6. The 40 most significantly regulated proteins by TT treatment (24 h) in TC-71 cells as fold-change relative to untreated control cells. (DOC 79 kb)

## Abbreviations

CD: Cyclodextrins
ER: Endoplasmatic reticulum
FDR: False discovery rate
ML-I: Mistletoe lectin I
NAC: N-acetylcysteine
TBST: Tris-buffered saline with Tween-20
TT: Triterpene acid-containing extract
LFQ: Label-free quantification

## References

[1] Society AC. American Cancer Society: Cancer Facts and Figures 2014. Am Cancer Soc. 2014. available online.

[2] von Levetzow C, Jiang X, Gwye Y, von Levetzow G, Hung L, Cooper A, Hsu JH-R, Lawlor ER (2011). Modeling initiation of Ewing sarcoma in human neural crest cells. PLoS One.

[3] Lin PP, Wang Y, Lozano G. Mesenchymal Stem Cells and the Origin of Ewing's Sarcoma. Sarcoma. 2011;2011:8. Article ID 276463. doi:10.1155/2011/276463. 10.1155/2011/276463 PMC2952797 20953407

[4] Paronetto MP (2013). Ewing sarcoma protein: a key player in human cancer. Int J Cell Biol.

[5] Mackintosh C, Madoz-Gurpide J (2013). Mining sarcomas by proteomics approaches: Ewing sarcoma on the spotlight. Recent Pat Biotechnol.

[6] Kelleher FC, Thomas DM (2012). Molecular pathogenesis and targeted therapeutics in Ewing sarcoma/primitive neuroectodermal tumours. Clinical Sarcoma Res.

[7] Amaral AT, Ordóñez JL, Otero-Motta AP, García-Domínguez DJ, Sevillano MV, de Álava E (2014). Innovative therapies in Ewing sarcoma. Adv Anat Pathol.

[8] Jung ML, Baudino S, Ribereau-Gayon G, Beck JP (1990). Characterization of cytotoxic proteins from mistletoe (*Viscum album* L.). Cancer Lett.

[9] Maletzki C, Linnebacher M, Savai R, Hobohm U (2013). Mistletoe lectin has a shiga toxin-like structure and should be combined with other toll-like receptor ligands in cancer therapy. Cancer Immunol Immunother.

[10] Park WB, Han SK, Lee MH, Han KH (1997). Isolation and characterization of lectins from stem and leaves of Korean mistletoe (*Viscum album* var.coloratum) by affinity chromatography. Arch Pharm Res.

[11] Strüh CM, Jäger S, Kersten A, Schempp CM, Scheffler A, Martin SF (2013). Triterpenoids amplify anti-tumoral effects of mistletoe extracts on murine B16.f10 melanoma in vivo. PLoS One.

[12] Tabiasco J, Pont F, Fournie JJ, Vercellone A (2002). Mistletoe viscotoxins increase natural killer cell-mediated cytotoxicity. Eur J Biochem.

[13] Jäger S, Trojan H, Kopp T, Laszczyk M, Scheffler A (2009). Pentacyclic Triterpene distribution in various plants–rich sources for a new group of multi-potent plant extracts. Molecules.

[14] Franz H, Ziska P, Kindt A (1981). Isolation and properties of three lectins from mistletoe (*Viscum album* L.). Biochem J.

[15] Orhan DD, Kupeli E, Yesilada E, Ergun F (2006). Anti-inflammatory and antinociceptive activity of flavonoids isolated from *Viscum album ssp. album*. Z Naturforsch C.

[16] Park YK, Do YR, Jang BC (2012). Apoptosis of K562 leukemia cells by Abnobaviscum F(R), a European mistletoe extract. Oncol Rep.

[17] Akl MR, Elsayed HE, Ebrahim HY, Haggag EG, Kamal AM, El Sayed KA (2014). 3-O-[N-(p-fluorobenzenesulfonyl)-carbamoyl]-oleanolic acid, a semisynthetic analog of oleanolic acid, induces apoptosis in breast cancer cells. Eur J Pharmacol.

[18] Strüh CM, Jäger S, Schempp CM, Scheffler A, Martin SF (2012). A novel Triterpene extract from mistletoe induces rapid apoptosis in Murine B16.F10 melanoma cells. Phytother Res.

[19] Tan Y, Yu R, Pezzuto JM (2003). Betulinic acid-induced programmed cell death in human melanoma cells involves Mitogen-activated protein Kinase activation. Clin Cancer Res.

[20] Guo G, Yao W, Zhang Q, Bo Y (2013). Oleanolic acid suppresses migration and invasion of malignant glioma cells by inactivating MAPK/ERK signaling pathway. PLoS One.

[21] Liu J, Wu N, Ma LN, Zhong JT, Liu G, Zheng LH, Lin XK (2014). p38 MAPK signaling mediates mitochondrial apoptosis in cancer cells induced by oleanolic acid. Asian Pac J Cancer Prev.

[22] Rabi T, Banerjee S (2008). Novel synthetic triterpenoid methyl 25-hydroxy-3-oxoolean-12-en-28-oate induces apoptosis through JNK and p38 MAPK pathways in human breast adenocarcinoma MCF-7 cells. Mol Carcinog.

[23] Lu Y, Zhu M, Chen W, Yin L, Zhu J, Chen N, Chen W (2014). Oleanolic acid induces apoptosis of MKN28 cells via AKT and JNK signaling pathways. Pharm Biol.

[24] Park R, Kim MS, So HS, Jung BH, Moon SR, Chung SY, Ko CB, Kim BR, Chung HT (2000). Activation of c-Jun N-terminal kinase 1 (JNK1) in mistletoe lectin II-induced apoptosis of human myeloleukemic U937 cells. Biochem Pharmacol.

[25] Kim JJ, Hwang YH, Kang KY, Kim I, Kim JB, Park JH, Yoo YC, Yee ST (2014). Enhanced dendritic cell maturation by the B-chain of Korean mistletoe lectin (KML-B), a novel TLR4 agonist. Int Immunopharmacol.

[26] Yang X, Jiang S, Liu Y, Zhang P, Xie S, Wang G (2012). Recombinant VAA-I from *Viscum album* induces apoptotic cell death of hepatocellular carcinoma SMMC7721 cells. Molecules.

[27] Lyu S-Y, Choi JH, Lee H-J, Park W-B, Kim GJ. Korean mistletoe lectin promotes proliferation and invasion of trophoblast cells through regulation of Akt signaling. Reprod Toxicol. 2013;39(0):33–9. 10.1016/j.reprotox.2013.03.011 23571125

[28] Choi SH, Lyu SY, Park WB (2004). Mistletoe lectin induces apoptosis and telomerase inhibition in human A253 cancer cells through dephosphorylation of Akt. Arch Pharm Res.

[29] Khil LY, Kim W, Lyu S, Park WB, Yoon JW, Jun HS (2007). Mechanisms involved in Korean mistletoe lectin-induced apoptosis of cancer cells. World J Gastroenterol.

[30] Gao X, Liu Y, Deeb D, Liu P, Liu A, Arbab AS, Gautam SC (2013). ROS mediate proapoptotic and antisurvival activity of oleanane triterpenoid CDDO-me in ovarian cancer cells. Anticancer Res.

[31] Deeb D, Gao X, Jiang H, Dulchavsky SA, Gautam SC (2009). Oleanane triterpenoid CDDO-me inhibits growth and induces apoptosis in prostate cancer cells by independently targeting pro-survival Akt and mTOR. Prostate.

[32] Deeb D, Gao X, Dulchavsky SA, Gautam SC (2007). CDDO-me induces apoptosis and inhibits Akt, mTOR and NF-kappaB signaling proteins in prostate cancer cells. Anticancer Res.

[33] Gao X, Deeb D, Hao J, Liu Y, Arbab AS, Dulchavsky SA, Gautam SC (2010). Synthetic Triterpenoids inhibit growth, induce apoptosis and suppress pro-survival Akt, mTOR and NF-κB signaling proteins in colorectal cancer cells. Anticancer Res.

[34] Zhou R, Zhang Z, Zhao L, Jia C, Xu S, Mai Q, Lu M, Huang M, Wang L, Wang X (2011). Inhibition of mTOR signaling by oleanolic acid contributes to its anti-tumor activity in osteosarcoma cells. J Orthop Res.

[35] Delebinski CI, Jäger S, Kemnitz-Hassanin K, Henze G, Lode HN, Seifert GJ (2012). A new development of triterpene acid-containing extracts from *Viscum album* L. displays synergistic induction of apoptosis in acute lymphoblastic leukaemia. Cell Prolif.

[36] Delebinski CI, Twardziok M, Kleinsimon S, Hoff F, Mulsow K, Rolff J, Jäger S, Eggert A, Seifert G (2015). A natural combination extract of *Viscum album* L. containing both Triterpene acids and Lectins is highly effective against AML *In Vivo*. PLoS One.

[37] Jaggy C, Musielski H, Urech K, Schaller G (1995). Quantitative determination of lectins in mistletoe preparations. Arzneimittelforschung.

[38] Twardziok M, Kleinsimon S, Rolff J, Jager S, Eggert A, Seifert G, Delebinski CI (2016). Multiple active compounds from *Viscum album* L. synergistically converge to promote apoptosis in Ewing sarcoma. PLoS One.

[39] Anders S, Huber W (2010). Differential expression analysis for sequence count data. Genome Biol.

[40] Benjamini Y, Drai D, Elmer G, Kafkafi N, Golani I (2001). Controlling the false discovery rate in behavior genetics research. Behav Brain Res.

[41] Livak KJ, Schmittgen TD (2001). Analysis of relative Gene expression data using real-time quantitative PCR and the 2−ΔΔCT method. Methods.

[42] Cox J, Mann M (2008). MaxQuant enables high peptide identification rates, individualized p.P.B.-range mass accuracies and proteome-wide protein quantification. Nat Biotechnol.

[43] Cox J, Hein MY, Luber CA, Paron I, Nagaraj N, Mann M (2014). Accurate proteome-wide label-free quantification by delayed normalization and maximal peptide ratio extraction, termed MaxLFQ. Mol Cell Proteomics.

[44] Subramanian A, Tamayo P, Mootha VK, Mukherjee S, Ebert BL, Gillette MA, Paulovich A, Pomeroy SL, Golub TR, Lander ES (2005). Gene set enrichment analysis: a knowledge-based approach for interpreting genome-wide expression profiles. Proc Natl Acad Sci U S A.

[45] Franceschini A, Szklarczyk D, Frankild S, Kuhn M, Simonovic M, Roth A, Lin J, Minguez P, Bork P, von Mering C (2013). STRING v9.1: protein-protein interaction networks, with increased coverage and integration. Nucleic Acids Res.

[46] Hoyer-Hansen M, Jaattela M (2007). Connecting endoplasmic reticulum stress to autophagy by unfolded protein response and calcium. Cell Death Differ.

[47] Heath-Engel HM, Chang NC, Shore GC (2008). The endoplasmic reticulum in apoptosis and autophagy: role of the BCL-2 protein family. Oncogene.

[48] Eggenschwiler J, Patrignani A, Wagner U, Rehrauer H, Schlapbach R, Rist L, Ramos MH, Viviani A (2006). Gene expression profiles of different breast cancer cells compared with their responsiveness to fermented mistletoe (*Viscum album* L.) extracts Iscador from oak (Quercus), pine (Pinus), white fir (Abies) and apple tree (Malus) in vitro. Arzneimittelforschung.

[49] Olsnes S, Stirpe F, Sandvig K, Pihl A (1982). Isolation and characterization of viscumin, a toxic lectin from *Viscum album* L. (mistletoe). J Biol Chem.

[50] Franz H, Kindt A, Ziska P, Bielka H, Benndorf R, Venker L (1982). The toxic A-chain of mistletoe lectin I: isolation and its effect on cell-free protein synthesis. Acta Biol Med Ger.

[51] Ye W, Nanga RP, Kang CB, Song JH, Song SK, Yoon HS (2006). Molecular characterization of the recombinant A-chain of a type II ribosome-inactivating protein (RIP) from *Viscum album* Coloratum and structural basis on its ribosome-inactivating activity and the sugar-binding properties of the B-chain. J Biochem Mol Biol.

[52] Barbieri L, Valbonesi P, Bondioli M, Alvarez ML, Dal Monte P, Landini MP, Stirpe F (2001). Adenine glycosylase activity in mammalian tissues: an equivalent of ribosome-inactivating proteins. FEBS Lett.

[53] Barbieri L, Valbonesi P, Bonora E, Gorini P, Bolognesi A, Stirpe F (1997). Polynucleotide: adenosine glycosidase activity of ribosome-inactivating proteins: effect on DNA, RNA and poly(a). Nucleic Acids Res.

[54] Kim MS, So HS, Lee KM, Park JS, Lee JH, Moon SK, Ryu DG, Chung SY, Jung BH, Kim YK (2000). Activation of caspase cascades in Korean mistletoe (*Viscum album* Var. Coloratum) lectin-II-induced apoptosis of human myeloleukemic U937 cells. Gen Pharmacol.

[55] Kim WH, Park WB, Gao B, Jung MH (2004). Critical role of reactive oxygen species and mitochondrial membrane potential in Korean mistletoe lectin-induced apoptosis in human hepatocarcinoma cells. Mol Pharmacol.

[56] Tesh VL (2012). Activation of cell stress response pathways by Shiga toxins. Cell Microbiol.

[57] Gonzalez TV, Farrant SA, Mantis NJ (2006). Ricin induces IL-8 secretion from human monocyte/macrophages by activating the p38 MAP kinase pathway. Mol Immunol.

[58] Lindauer M, Wong J, Magun B (2010). Ricin toxin activates the NALP3 Inflammasome. Toxins.

[59] Chen J-Y, Zhang L, Zhang H, Su L, Qin L-P (2014). Triggering of p38 MAPK and JNK signaling is important for Oleanolic acid-induced apoptosis via the mitochondrial death pathway in hypertrophic scar fibroblasts. Phytother Res.

[60] Auletta JJ, Alabran JL, Kim B-G, Meyer CJ, Letterio JJ (2010). The synthetic Triterpenoid, CDDO-me, modulates the Proinflammatory response to in vivo Lipopolysaccharide challenge. J Interf Cytokine Res.

[61] Petronelli A, Pannitteri G, Testa U (2009). Triterpenoids as new promising anticancer drugs. Anti-Cancer Drugs.

[62] Pae HO, Oh GS, Kim NY, Shin MK, Lee HS, Yun YG, Oh H, Kim YM, Chung HT (2001). Roles of extracellular signal-regulated kinase and p38 mitogen-activated protein kinase in apoptosis of human monoblastic leukemia U937 cells by lectin-II isolated from Korean mistletoe. In Vitr Mol Toxicol.

[63] Kim I, Shu CW, Xu W, Shiau CW, Grant D, Vasile S, Cosford ND, Reed JC (2009). Chemical biology investigation of cell death pathways activated by endoplasmic reticulum stress reveals cytoprotective modulators of ASK1. J Biol Chem.

[64] Urano F, Wang X, Bertolotti A, Zhang Y, Chung P, Harding HP, Ron D (2000). Coupling of stress in the ER to activation of JNK protein kinases by transmembrane protein kinase IRE1. Science.

[65] Nishitoh H, Matsuzawa A, Tobiume K, Saegusa K, Takeda K, Inoue K, Hori S, Kakizuka A, Ichijo H (2002). ASK1 is essential for endoplasmic reticulum stress-induced neuronal cell death triggered by expanded polyglutamine repeats. Genes Dev.

[66] Cullinan SB, Diehl JA (2006). Coordination of ER and oxidative stress signaling: the PERK/Nrf2 signaling pathway. Int J Biochem Cell Biol.

[67] Wang M, Wey S, Zhang Y, Ye R, Lee AS (2009). Role of the unfolded protein response regulator GRP78/BiP in development, cancer, and neurological disorders. Antioxid Redox Signal.

[68] Jäger R, Bertrand MJM, Gorman AM, Vandenabeele P, Samali A (2012). The unfolded protein response at the crossroads of cellular life and death during endoplasmic reticulum stress. Biol Cell.

[69] Chakrabarti A, Chen AW, Varner JD (2011). A review of the mammalian unfolded protein response. Biotechnol Bioeng.

[70] Horrix C, Raviv Z, Flescher E, Voss C, Berger MR (2011). Plant ribosome-inactivating proteins type II induce the unfolded protein response in human cancer cells. Cell Mol Life Sci.

[71] Zhao Y, Tian T, Huang T, Nakajima S, Saito Y, Takahashi S, Yao J, Paton AW, Paton JC, Kitamura M (2011). Subtilase Cytotoxin activates MAP Kinases through PERK and IRE1 branches of the unfolded protein response. Toxicol Sci.

[72] Fribley AM, Miller JR, Brownell AL, Garshott DM, Zeng Q, Reist TE, Narula N, Cai P, Xi Y, Callaghan MU (2015). Celastrol induces unfolded protein response-dependent cell death in head and neck cancer. Exp Cell Res.

[73] Won S-J, Ki YS, Chung K-S, Choi J-H, Bae KH, Lee K-T (2010). 3&alpha;,23-isopropylidenedioxyolean-12-en-27-oic acid, a triterpene isolated from aceriphyllum rossii, induces apoptosis in human cervical cancer HeLa cells through mitochondrial dysfunction and endoplasmic reticulum stress. Biol Pharm Bull.

[74] Xu T, Pang Q, Zhou D, Zhang A, Luo S, Wang Y, Yan X (2014). Proteomic investigation into betulinic acid-induced apoptosis of human cervical cancer HeLa cells. PLoS One.

[75] Zou W, Yue P, Khuri FR, Sun S-Y (2008). Coupling of endoplasmic reticulum stress to CDDO-me–induced up-regulation of death receptor 5 via a CHOP–dependent mechanism involving JNK activation. Cancer Res.

[76] Schleicher SM, Moretti L, Varki V, Lu B (2010). Progress in the unraveling of the endoplasmic reticulum stress/autophagy pathway and cancer: implications for future therapeutic approaches. Drug Resist Updat.

[77] Appenzeller-Herzog C, Hall MN (2012). Bidirectional crosstalk between endoplasmic reticulum stress and mTOR signaling. Trends Cell Biol.

[78] Shen S, Zhang Y, Zhang R, Tu X, Gong X (2014). Ursolic acid induces autophagy in U87MG cells via ROS-dependent endoplasmic reticulum stress. Chem Biol Interact.

[79] Zhao C, Yin S, Dong Y, Guo X, Fan L, Ye M, Hu H (2013). Autophagy-dependent EIF2AK3 activation compromises ursolic acid-induced apoptosis through upregulation of MCL1 in MCF-7 human breast cancer cells. Autophagy.

[80] Yore MM, Kettenbach AN, Sporn MB, Gerber SA, Liby KT (2011). Proteomic analysis shows synthetic Oleanane Triterpenoid binds to mTOR. PLoS One.

[81] Liu J, Zheng L, Ma L, Wang B, Zhao Y, Wu N, Liu G, Lin X (2014). Oleanolic acid inhibits proliferation and invasiveness of Kras-transformed cells via autophagy. J Nutr Biochem.

[82] Liu J, Zheng L, Zhong J, Wu N, Liu G, Lin X (2014). Oleanolic acid induces protective autophagy in cancer cells through the JNK and mTOR pathways. Oncol Rep.

[83] Lisiak N, Paszel-Jaworska A, Bednarczyk-Cwynar B, Zaprutko L, Kaczmarek M, Rybczynska M (2014). Methyl 3-hydroxyimino-11-oxoolean-12-en-28-oate (HIMOXOL), a synthetic oleanolic acid derivative, induces both apoptosis and autophagy in MDA-MB-231 breast cancer cells. Chem Biol Interact.

[84] Yang L-j, Chen Y, He J, Yi S, Wen L, Zhao J, Zhang B-p, Cui G-h (2012). Betulinic acid inhibits autophagic flux and induces apoptosis in human multiple myeloma cells in vitro. Acta Pharmacol Sin.

[85] Choi JH, Lyu SY, Lee HJ, Jung J, Park WB, Kim GJ (2012). Korean mistletoe lectin regulates self-renewal of placenta-derived mesenchymal stem cells via autophagic mechanisms. Cell Prolif.

