# Supplementary figures and images for: Transcriptomic and proteomic insight into the effects of a defined European mistletoe extract in Ewing sarcoma cells reveals cellular stress responses
Source: BMC Complement Altern Med. 2017 Apr 28;17:237. doi: 10.1186/s12906-017-1715-2 (PMC5410041; doi:10.1186/s12906-017-1715-2)

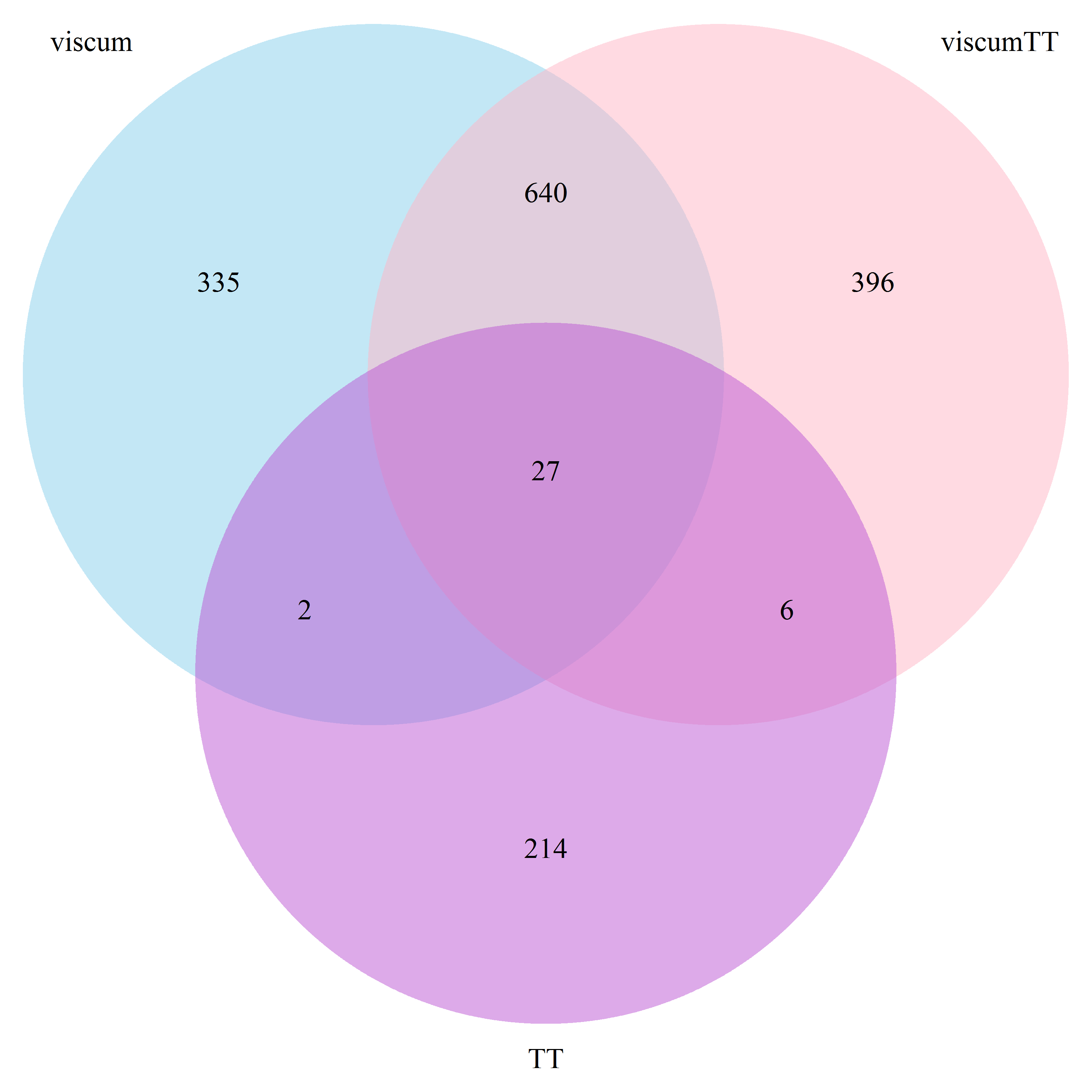

Supplement: Supplementary file 1 — Differentially expressed genes after treatment with viscum, TT or viscumTT in TC-71 cells. TC-71 cells were incubated for 24 h with viscumTT, viscum or TT in ~ IC50 concentrations in reference to untreated control cells followed by one mRNA sequencing experiment. Normalisation and identification of differentially expressed genes was performed using DE-Seq software (Bioconductor open source software) to calculate fold-change relative to untreated control cells, false discovery rate and p value using the negative binomial distribution, with p ≤ 0.05 considered as significant. Venn diagram displays uniquely and commonly deregulated genes by the extracts. [file 12906_2017_1715_MOESM1_ESM.tiff]
