# Supplementary material for: Transcriptomic and proteomic insight into the effects of a defined European mistletoe extract in Ewing sarcoma cells reveals cellular stress responses
Source: BMC Complement Altern Med. 2017 Apr 28;17:237. doi: 10.1186/s12906-017-1715-2 (PMC5410041; doi:10.1186/s12906-017-1715-2)
Supplement: Supplementary file 2 — The 40 most significantly regulated genes by viscumTT treatment (24 h) in TC-71 cells as fold-change relative to untreated control cells. (DOCX 14 kb) [file 12906_2017_1715_MOESM2_ESM.docx]

**Table S1.** The 40 most significantly regulated genes by viscumTT treatment (24 h) in TC-71 cells as fold-change relative to untreated control cells.

| **ENSTID** | **Gene** | **p-Value** | **Control** | **ViscumTT** | **Fold-change** |
| --- | --- | --- | --- | --- | --- |
| ENST00000307407 | IL8 | 7.32x10^-8 | 4.23 | 5983.00 | 1414.59 |
| ENST00000357727 | CREB5 | 2.91x10^-7 | 0.00 | 1084.00 | NA |
| ENST00000377507 | TNFRSF9 | 3.11x10^-7 | 0.00 | 1072.00 | NA |
| ENST00000482692 | CREB5 | 3.73x10^-6 | 0.00 | 439.00 | NA |
| ENST00000249330 | VGF | 4.04x10^-6 | 0.00 | 427.00 | NA |
| ENST00000371222 | JUN | 3.12x10^-5 | 206.19 | 22624.00 | 109.73 |
| ENST00000371826 | IFIT2 | 1.77x10^-4 | 1.06 | 176.00 | 166.45 |
| ENST00000409458 | GPNMB | 1.83x10^-4 | 2.11 | 235.00 | 111.12 |
| ENST00000445961 | RPS9 | 3.21x10^-4 | 0.00 | 91.00 | NA |
| ENST00000414584 | LINC00152 | 3.79x10^-4 | 1.06 | 133.00 | 125.78 |
| ENST00000223095 | SERPINE1 | 4.00x10^-4 | 0.00 | 82.00 | NA |
| ENST00000264930 | SLC12A7 | 4.16x10^-4 | 22.20 | 1029.00 | 46.34 |
| ENST00000257570 | OASL | 4.52x10^-4 | 1.06 | 124.00 | 117.27 |
| ENST00000424148 | KRTAP5-AS1 | 5.15x10^-4 | 0.00 | 76.00 | NA |
| ENST00000326577 | TNFRSF12A | 5.35x10^-4 | 2.11 | 163.00 | 77.08 |
| ENST00000369165 | HIST2H4A | 5.71x10^-4 | 1.06 | 115.00 | 108.76 |
| ENST00000304338 | PPP4R4 | 5.83x10^-4 | 3.17 | 200.00 | 63.05 |
| ENST00000394684 | SGMS2 | 6.59x10^-4 | 27.49 | 1049.00 | 38.16 |
| ENST00000474904 | GPATCH4 | 6.65x10^-4 | 1.06 | 109.00 | 103.09 |
| ENST00000239938 | EGR1 | 7.35x10^-4 | 540.32 | 18438.00 | 34.12 |
| ENST00000200453 | PPP1R15A | 7.87x10^-4 | 432.46 | 14397.00 | 33.29 |
| ENST00000429295 | MRPL23 | 8.81x10^-4 | 9.52 | 370.00 | 38.88 |
| ENST00000550903 | PPP1R12A | 8.92x10^-4 | 0.00 | 61.00 | NA |
| ENST00000377831 | HIST1H3D | 9.32x10^-4 | 1.06 | 96.00 | 90.79 |
| ENST00000304218 | HIST1H1E | 9.33x10^-4 | 6.34 | 263.00 | 41.46 |
| ENST00000346473 | DDIT3 | 9.43x10^-4 | 29.61 | 982.00 | 33.17 |
| ENST00000533621 | BCLAF1 | 1.04x10^-3 | 0.00 | 58.00 | NA |
| ENST00000295927 | PTX3 | 1.06x10^-3 | 0.00 | 61.00 | NA |
| ENST00000259874 | IER3 | 1.07x10^-3 | 2.11 | 126.00 | 59.58 |
| ENST00000592209 | WASH5P | 1.07x10^-3 | 0.00 | 57.00 | NA |
| ENST00000461675 | MRPS31 | 1.21x10^-3 | 0.00 | 55.00 | NA |
| ENST00000542735 | DND1 | 1.23x10^-3 | 0.00 | 56.00 | NA |
| ENST00000506674 | MRPL1 | 1.23x10^-3 | 6.34 | 237.00 | 37.36 |
| ENST00000538682 | SLC3A2 | 1.47x10^-3 | 7.40 | 250.00 | 33.78 |
| ENST00000296252 | LIPH | 1.55x10^-3 | 1.06 | 79.00 | 74.71 |
| ENST00000423282 | STAT1 | 1.58x10^-3 | 1.06 | 79.00 | 74.71 |
| ENST00000380659 | TLR7 | 1.59x10^-3 | 3.17 | 135.00 | 42.56 |
| ENST00000419736 | MIR4435-1HG | 1.63x10^-3 | 0.00 | 50.00 | NA |
| ENST00000564543 | RP11-20I23.1 | 1.66x10^-3 | 99.39 | 2.00 | 0.02 |
| ENST00000173785 | KLF6 | 1.68x10^-3 | 0.00 | 50.00 | NA |

* p ≤ 0.05, N/A not available
