# Supplementary material for: Transcriptomic and proteomic insight into the effects of a defined European mistletoe extract in Ewing sarcoma cells reveals cellular stress responses
Source: BMC Complement Altern Med. 2017 Apr 28;17:237. doi: 10.1186/s12906-017-1715-2 (PMC5410041; doi:10.1186/s12906-017-1715-2)
Supplement: Supplementary file 3 — The 40 most significantly regulated genes by viscum treatment (24 h) in TC-71 cells as fold-change relative to untreated control cells. (DOC 78 kb) [file 12906_2017_1715_MOESM3_ESM.doc]

**Table S2.** The 40 most significantly regulated genes by viscum treatment (24 h) in TC-71 cells as fold-change relative to untreated control cells.

| **ENSTID** | **Gene** | **p-Value** | **Control** | **Viscum** | **Fold-change** |
| --- | --- | --- | --- | --- | --- |
| ENST00000357727 | CREB5 | 1.32x10^-7 | 0.00 | 1451.99 | NA |
| ENST00000482692 | CREB5 | 7.45x10^-6 | 0.00 | 343.33 | NA |
| ENST00000249330 | VGF | 8.98x10^-6 | 0.00 | 320.98 | NA |
| ENST00000371222 | JUN | 5.63x10^-5 | 206.19 | 18368.89 | 89.09 |
| ENST00000409458 | GPNMB | 3.06x10^-4 | 2.11 | 194.91 | 92.17 |
| ENST00000414584 | LINC00152 | 3.07x10^-4 | 1.06 | 144.84 | 136.98 |
| ENST00000223095 | SERPINE1 | 4.02x10^-4 | 0.00 | 82.26 | NA |
| ENST00000239938 | EGR1 | 4.95x10^-4 | 540.32 | 21507.12 | 39.80 |
| ENST00000215885 | PLA2G3 | 5.36x10^-4 | 998.16 | 24.14 | 0.02 |
| ENST00000550903 | PPP1R12A | 5.84x10^-4 | 0.00 | 72.42 | NA |
| ENST00000394684 | SGMS2 | 6.46x10^-4 | 27.49 | 1063.96 | 38.70 |
| ENST00000592209 | WASH5P | 6.98x10^-4 | 0.00 | 67.95 | NA |
| ENST00000369165 | HIST2H4A | 7.56x10^-4 | 1.06 | 103.71 | 98.09 |
| ENST00000289352 | HIST1H4H | 8.81x10^-4 | 27.49 | 947.73 | 34.47 |
| ENST00000346473 | DDIT3 | 9.51x10^-4 | 29.61 | 985.28 | 33.28 |
| ENST00000296252 | LIPH | 9.61x10^-4 | 1.06 | 95.67 | 90.48 |
| ENST00000533621 | BCLAF1 | 9.68x10^-4 | 0.00 | 59.90 | NA |
| ENST00000429295 | MRPL23 | 9.79x10^-4 | 9.52 | 357.63 | 37.58 |
| ENST00000304218 | HIST1H1E | 1.00x10^-3 | 6.34 | 257.50 | 40.59 |
| ENST00000474904 | GPATCH4 | 1.04x10^-3 | 1.06 | 92.09 | 87.09 |
| ENST00000403537 | THUMPD2 | 1.07x10^-3 | 0.00 | 58.12 | NA |
| ENST00000464213 | CD36 | 1.13x10^-3 | 1.06 | 91.20 | 86.25 |
| ENST00000532854 | BMS1P2 | 1.23x10^-3 | 2.11 | 118.02 | 55.81 |
| ENST00000424148 | KRTAP5-AS1 | 1.25x10^-3 | 0.00 | 53.64 | NA |
| ENST00000489615 | RABGAP1L | 1.29x10^-3 | 4.23 | 176.13 | 41.64 |
| ENST00000506674 | MRPL1 | 1.29x10^-3 | 6.34 | 234.25 | 36.92 |
| ENST00000569313 | CTD-2636A23.2 | 1.36x10^-3 | 96.22 | 1.79 | 0.02 |
| ENST00000570279 | CFDP1 | 1.47x10^-3 | 1.06 | 82.26 | 77.79 |
| ENST00000377831 | HIST1H3D | 1.52x10^-3 | 1.06 | 79.57 | 75.26 |
| ENST00000200453 | PPP1R15A | 1.54x10^-3 | 432.46 | 11251.11 | 26.02 |
| ENST00000490692 | PCSK9 | 1.60x10^-3 | 66.61 | 0.89 | 0.01 |
| ENST00000461675 | MRPS31 | 1.61x10^-3 | 0.00 | 49.17 | NA |
| ENST00000556330 | SRSF5 | 1.84x10^-3 | 7.40 | 232.46 | 31.41 |
| ENST00000446547 | RAB5A | 1.86x10^-3 | 4.23 | 152.89 | 36.15 |
| ENST00000481490 | FSBP | 1.89x10^-3 | 6.34 | 202.96 | 31.99 |
| ENST00000554437 | SCFD1 | 1.90x10^-3 | 0.00 | 47.39 | NA |
| ENST00000317216 | EGR3 | 1.93x10^-3 | 10.57 | 303.99 | 28.75 |
| ENST00000506974 | NDUFS4 | 1.99x10^-3 | 10.57 | 298.62 | 28.24 |
| ENST00000369317 | TXNIP | 2.08x10^-3 | 118.43 | 2783.27 | 23.50 |
| ENST00000435030 | KIF5C | 2.10x10^-3 | 2.11 | 96.56 | 45.66 |

* p ≤ 0.05, N/A not available
