# Supplementary material for: Transcriptomic and proteomic insight into the effects of a defined European mistletoe extract in Ewing sarcoma cells reveals cellular stress responses
Source: BMC Complement Altern Med. 2017 Apr 28;17:237. doi: 10.1186/s12906-017-1715-2 (PMC5410041; doi:10.1186/s12906-017-1715-2)
Supplement: Supplementary file 4 — The 40 most significantly regulated genes by TT treatment (24 h) in TC-71 cells as fold-change relative to untreated control cells. (DOC 77 kb) [file 12906_2017_1715_MOESM4_ESM.doc]

**Table S3.** The 40 most significantly regulated genes by TT treatment (24 h) in TC-71 cells as fold-change relative to untreated control cells.

| **ENSTID** | **Gene** | **p-Value** | **Control** | **TT** | **Fold-change** |
| --- | --- | --- | --- | --- | --- |
| ENST00000395676 | KLHDC7B | 4.06x10^-7 | 2.11 | 2127.00 | 1005.80 |
| ENST00000252809 | GDF15 | 2.22x10^-6 | 1.06 | 853.00 | 806.72 |
| ENST00000441316 | RP4-756G23.5 | 3.65x10^-6 | 0.00 | 451.00 | NA |
| ENST00000324873 | NUPR1 | 1.77x10^-5 | 2.11 | 556.00 | 262.92 |
| ENST00000407325 | GPR1 | 4.52x10^-5 | 1.06 | 295.00 | 278.99 |
| ENST00000496593 | RPLP0P2 | 1.04x10^-4 | 1.06 | 220.00 | 208.06 |
| ENST00000266646 | INHBE | 1.11x10^-4 | 16.92 | 1306.00 | 77.20 |
| ENST00000450895 | AP000322.53 | 1.44x10^-4 | 0.00 | 125.00 | NA |
| ENST00000416826 | RP11-125B21.2 | 1.98x10^-4 | 1.06 | 175.00 | 165.50 |
| ENST00000218867 | SGCG | 2.23x10^-4 | 1.06 | 168.00 | 158.88 |
| ENST00000257570 | OASL | 2.38x10^-4 | 1.06 | 158.00 | 149.43 |
| ENST00000572856 | DLGAP1-AS2 | 2.73x10^-4 | 0.00 | 97.00 | NA |
| ENST00000426475 | AC007405.6 | 3.06x10^-4 | 0.00 | 96.00 | NA |
| ENST00000273353 | MYH15 | 3.20x10^-4 | 16.92 | 888.00 | 52.49 |
| ENST00000582155 | NFE2L1 | 3.48x10^-4 | 0.00 | 92.00 | NA |
| ENST00000409652 | APOL6 | 3.79x10^-4 | 0.00 | 89.00 | NA |
| ENST00000371930 | ANKRD22 | 3.82x10^-4 | 0.00 | 89.00 | NA |
| ENST00000371826 | IFIT2 | 3.95x10^-4 | 1.06 | 130.00 | 122.95 |
| ENST00000445281 | OSTN | 4.15x10^-4 | 2.11 | 181.00 | 85.59 |
| ENST00000265598 | LAMP3 | 4.66x10^-4 | 3.17 | 217.00 | 68.41 |
| ENST00000338333 | FBLL1 | 7.12x10^-4 | 2.11 | 149.00 | 70.46 |
| ENST00000512913 | SUB1 | 9.31x10^-4 | 0.00 | 65.00 | NA |
| ENST00000405493 | RAPGEF3 | 1.01x10^-3 | 0.00 | 63.00 | NA |
| ENST00000409458 | GPNMB | 1.21x10^-3 | 2.11 | 115.00 | 54.38 |
| ENST00000415913 | IDH1 | 1.26x10^-3 | 7.40 | 270.00 | 36.48 |
| ENST00000288976 | PTPDC1 | 1.65x10^-3 | 0.00 | 53.00 | NA |
| ENST00000409039 | DNAH10 | 1.73x10^-3 | 6.34 | 213.00 | 33.57 |
| ENST00000415282 | IDH1 | 1.74x10^-3 | 11.63 | 344.00 | 29.58 |
| ENST00000303965 | ARAP2 | 1.82x10^-3 | 0.00 | 50.00 | NA |
| ENST00000580037 | NFE2L1 | 1.91x10^-3 | 0.00 | 50.00 | NA |
| ENST00000519505 | ESRP1 | 1.94x10^-3 | 0.00 | 50.00 | NA |
| ENST00000536222 | NFE2L1 | 2.76x10^-3 | 1.06 | 68.00 | 64.31 |
| ENST00000493793 | EPS15 | 2.95x10^-3 | 6.34 | 175.00 | 27.58 |
| ENST00000434245 | RP11-495P10.8 | 3.18x10^-3 | 2.11 | 86.00 | 40.67 |
| ENST00000547303 | DDIT3 | 3.20x10^-3 | 4.23 | 122.00 | 28.85 |
| ENST00000287713 | NMNAT2 | 3.27x10^-3 | 0.00 | 41.00 | NA |
| ENST00000264474 | CSTA | 3.66x10^-3 | 6.34 | 161.00 | 25.38 |
| ENST00000454170 | ESRP1 | 4.10x10^-3 | 0.00 | 38.00 | NA |
| ENST00000361488 | FAM110B | 4.40x10^-3 | 4.23 | 113.00 | 26.72 |
| ENST00000380659 | TLR7 | 4.56x10^-3 | 3.17 | 89.00 | 28.06 |

* p ≤ 0.05, N/A not available
