# Supplementary material for: Transcriptomic and proteomic insight into the effects of a defined European mistletoe extract in Ewing sarcoma cells reveals cellular stress responses
Source: BMC Complement Altern Med. 2017 Apr 28;17:237. doi: 10.1186/s12906-017-1715-2 (PMC5410041; doi:10.1186/s12906-017-1715-2)
Supplement: Supplementary file 5 — The 40 most significantly regulated proteins by viscumTT treatment (24 h) in TC-71 cells as fold-change relative to untreated control cells (DOC 79 kb) [file 12906_2017_1715_MOESM5_ESM.doc]

**Table S4:** The 40 most significantly regulated proteins by viscumTT treatment (24 h) in TC-71 cells as fold-change relative to untreated control cells.

| **Downregulated** |  |  |  | **Upregulated** |  |  |
| --- | --- | --- | --- | --- | --- | --- |
| **protein** | **Fold-change** | **PEP** |  | **protein** | **Fold-change** | **PEP** |
| PKP1 | 0.07 | <0.001 |  | RRP12 | 2.69 | <0.001 |
| RPL39P5 | 0.15 | <0.001 |  | DDX39A | 2.69 | <0.001 |
| HIST1H2AC | 0.18 | <0.001 |  | GNB2L1 | 2.70 | <0.001 |
| RPS28 | 0.19 | <0.001 |  | OSBPL8 | 2.74 | <0.001 |
| HIST1H3A | 0.20 | 0.00 |  | CLTC | 2.77 | 0.00 |
| CKS2 | 0.22 | <0.001 |  | KMO | 2.79 | <0.001 |
| EDF1 | 0.23 | <0.001 |  | DIS3 | 2.83 | <0.001 |
| CHCHD2 | 0.26 | <0.001 |  | DHCR24 | 2.85 | <0.001 |
| CNBP | 0.27 | <0.001 |  | CS | 2.85 | <0.001 |
| POLR2D | 0.27 | <0.001 |  | ACBD3 | 2.90 | <0.001 |
| RPL10 | 0.27 | <0.001 |  | TMEM41B | 2.91 | <0.001 |
| BRD2 | 0.27 | <0.001 |  | SEL1L | 2.91 | <0.001 |
| NDUFB10 | 0.27 | <0.001 |  | PRPF8 | 2.93 | <0.001 |
| TPX2 | 0.28 | <0.001 |  | RAB2A | 2.94 | <0.001 |
| CETN2 | 0.28 | <0.001 |  | PRPS1 | 2.98 | <0.001 |
| CD248 | 0.29 | <0.001 |  | NAA25 | 3.01 | <0.001 |
| NOB1 | 0.30 | <0.001 |  | USP7 | 3.03 | <0.001 |
| UBE2C | 0.31 | <0.001 |  | MARCKS | 3.04 | <0.001 |
| PDCL | 0.32 | <0.001 |  | PSMB4 | 3.07 | <0.001 |
| CCDC159 | 0.33 | 1.37x10^-03 |  | SLC25A3 | 3.08 | <0.001 |
| ELF1 | 0.33 | <0.001 |  | PRKDC | 3.13 | 0.00 |
| MLLT11 | 0.33 | <0.001 |  | APEH | 3.21 | <0.001 |
| CDCA8 | 0.33 | <0.001 |  | ENOPH1 | 3.29 | <0.001 |
| POLR2L | 0.33 | <0.001 |  | TTLL12 | 3.30 | <0.001 |
| LLPH | 0.33 | <0.001 |  | PTMA | 3.32 | <0.001 |
| RBM19 | 0.34 | <0.001 |  | PSMB5 | 3.34 | <0.001 |
| KIAA0101 | 0.34 | <0.001 |  | OTUB1 | 3.40 | <0.001 |
| CCDC137 | 0.35 | <0.001 |  | RAB11B | 3.42 | <0.001 |
| NTPCR | 0.35 | <0.001 |  | DDB1 | 3.45 | <0.001 |
| TRMT112 | 0.35 | <0.001 |  | TM9SF2 | 3.51 | <0.001 |
| CRIP1 | 0.35 | <0.001 |  | DIAPH1 | 3.52 | <0.001 |
| POU3F1 | 0.36 | <0.001 |  | BCAP31 | 3.55 | <0.001 |
| LYRM7 | 0.36 | <0.001 |  | DYNC1H1 | 3.55 | <0.001 |
| HIBADH | 0.37 | <0.001 |  | MAP2K1 | 3.63 | <0.001 |
| COA4 | 0.37 | <0.001 |  | IPO7 | 3.66 | <0.001 |
| MESDC2 | 0.37 | <0.001 |  | HMGN3 | 3.86 | <0.001 |
| SRP14 | 0.37 | <0.001 |  | FTL | 4.07 | <0.001 |
| FAM207A | 0.38 | <0.001 |  | APOB | 4.07 | <0.001 |
| NDUFA4 | 0.38 | <0.001 |  | TBL3 | 4.08 | <0.001 |
| PPP4R2 | 0.38 | <0.001 |  | SPCS2 | 4.16 | <0.001 |

*FDR ≤ 0.01, PEP = posterior error probability
