# Supplementary material for: Transcriptomic and proteomic insight into the effects of a defined European mistletoe extract in Ewing sarcoma cells reveals cellular stress responses
Source: BMC Complement Altern Med. 2017 Apr 28;17:237. doi: 10.1186/s12906-017-1715-2 (PMC5410041; doi:10.1186/s12906-017-1715-2)
Supplement: Supplementary file 6 — The 40 most significantly regulated proteins by viscum treatment (24 h) in TC-71 cells as fold-change relative to untreated control cells. (DOC 79 kb) [file 12906_2017_1715_MOESM6_ESM.doc]

**Table S5:** The 40 most significantly regulated proteins by viscum treatment (24 h) in TC-71 cells as fold-change relative to untreated control cells.

| **Downregulated** |  |  |  | **Upregulated** |  |  |
| --- | --- | --- | --- | --- | --- | --- |
| **protein** | **Fold-change** | **PEP** |  | **protein** | **Fold-change** | **PEP** |
| PKP1 | 0.12 | <0.001 |  | MARCKS | 2.44 | <0.001 |
| ELF1 | 0.18 | <0.001 |  | ARL8B | 2.44 | <0.001 |
| CETN2 | 0.18 | <0.001 |  | IPO5 | 2.45 | 0 |
| CKS2 | 0.18 | <0.001 |  | DDOST | 2.46 | <0.001 |
| UBE2T | 0.19 | <0.001 |  | S100A13 | 2.47 | <0.001 |
| RPS28 | 0.20 | <0.001 |  | DDX3X | 2.47 | <0.001 |
| TYMS | 0.20 | <0.001 |  | INTS3 | 2.47 | <0.001 |
| HIST1H2AC | 0.21 | <0.001 |  | ENOPH1 | 2.49 | <0.001 |
| CCDC159 | 0.23 | 0.0013738 |  | PSMA3 | 2.50 | <0.001 |
| RPL39P5 | 0.26 | <0.001 |  | FNDC3A | 2.51 | <0.001 |
| CCNB1 | 0.26 | <0.001 |  | EIF2S1 | 2.51 | <0.001 |
| LTV1 | 0.27 | <0.001 |  | PSMB5 | 2.54 | <0.001 |
| MRPS6 | 0.27 | <0.001 |  | TARS | 2.56 | <0.001 |
| MLLT11 | 0.28 | <0.001 |  | TROVE2 | 2.58 | <0.001 |
| LLPH | 0.28 | <0.001 |  | RPN2 | 2.60 | <0.001 |
| SAFB2 | 0.28 | <0.001 |  | OTUB1 | 2.62 | <0.001 |
| RPL10 | 0.30 | <0.001 |  | DIAPH1 | 2.62 | <0.001 |
| PDCL | 0.30 | <0.001 |  | THOP1 | 2.69 | <0.001 |
| CDCA8 | 0.31 | <0.001 |  | PSMB4 | 2.70 | <0.001 |
| CNBP | 0.31 | <0.001 |  | PTMA | 2.71 | <0.001 |
| FAM207A | 0.31 | <0.001 |  | TTLL12 | 2.73 | <0.001 |
| HIST1H3A | 0.31 | 0 |  | RAB11B | 2.73 | <0.001 |
| TOMM6 | 0.31 | <0.001 |  | KDSR | 2.73 | <0.001 |
| USP36 | 0.32 | <0.001 |  | OGDH | 2.89 | <0.001 |
| CRIP1 | 0.32 | <0.001 |  | UAP1 | 2.91 | <0.001 |
| UBE2C | 0.33 | <0.001 |  | NAA25 | 2.95 | <0.001 |
| CDC42SE2 | 0.33 | <0.001 |  | MAP2K1 | 2.97 | <0.001 |
| FKBP11 | 0.33 | <0.001 |  | AP2B1 | 3.00 | <0.001 |
| BRD2 | 0.34 | <0.001 |  | DDB1 | 3.01 | <0.001 |
| COX7C | 0.34 | <0.001 |  | COPB2 | 3.04 | <0.001 |
| MKI67 | 0.35 | <0.001 |  | GNB2L1 | 3.05 | <0.001 |
| MTHFD2 | 0.36 | <0.001 |  | SF3B3 | 3.07 | <0.001 |
| FDX1 | 0.36 | <0.001 |  | CLTC | 3.12 | 0 |
| HIST2H3A | 0.36 | 0 |  | NUCKS1 | 3.13 | <0.001 |
| SELH | 0.36 | <0.001 |  | TBCD | 3.19 | <0.001 |
| NDUFB6 | 0.37 | <0.001 |  | PRKDC | 3.23 | 0 |
| BTF3 | 0.37 | 0 |  | IPO7 | 3.24 | <0.001 |
| HSPB1 | 0.37 | 0 |  | ASNS | 3.28 | <0.001 |
| UBE2S | 0.37 | <0.001 |  | VDAC3 | 3.31 | <0.001 |
| MED15 | 0.38 | <0.001 |  | KMO | 3.42 | <0.001 |

*FDR ≤ 0.01, PEP = posterior error probability
