# Supplementary material for: Transcriptomic and proteomic insight into the effects of a defined European mistletoe extract in Ewing sarcoma cells reveals cellular stress responses
Source: BMC Complement Altern Med. 2017 Apr 28;17:237. doi: 10.1186/s12906-017-1715-2 (PMC5410041; doi:10.1186/s12906-017-1715-2)
Supplement: Supplementary file 7 — The 40 most significantly regulated proteins by TT treatment (24 h) in TC-71 cells as fold-change relative to untreated control cells. (DOC 79 kb) [file 12906_2017_1715_MOESM7_ESM.doc]

**Table S6:** The 40 most significantly regulated proteins by TT treatment (24 h) in TC-71 cells as fold-change relative to untreated control cells.

| **Downregulated** |  |  |  | **Upregulated** |  |  |
| --- | --- | --- | --- | --- | --- | --- |
| **protein** | **Fold-change** | **PEP** |  | **protein** | **Fold-change** | **PEP** |
| MKI67 | 0.08 | <0.001 |  | STX6 | 1.95 | <0.001 |
| KIAA0101 | 0.08 | <0.001 |  | FNDC3A | 1.96 | <0.001 |
| TPX2 | 0.19 | <0.001 |  | ANXA1 | 1.96 | 0 |
| UBE2C | 0.22 | <0.001 |  | ESYT1 | 1.97 | <0.001 |
| CDCA8 | 0.22 | <0.001 |  | IDI1 | 1.97 | <0.001 |
| INCENP | 0.24 | <0.001 |  | ROBO2 | 1.98 | <0.001 |
| RPL39P5 | 0.26 | <0.001 |  | WARS | 1.99 | 0.00 |
| MRFAP1 | 0.27 | <0.001 |  | SLC3A2 | 1.99 | 0.00 |
| KIF11 | 0.31 | <0.001 |  | PSMB6 | 2.00 | <0.001 |
| RIC8A | 0.32 | <0.001 |  | DTD1 | 2.04 | <0.001 |
| EP400 | 0.35 | <0.001 |  | ZFPL1 | 2.04 | <0.001 |
| TMSB15B | 0.35 | <0.001 |  | MVD | 2.04 | <0.001 |
| KPNA2 | 0.35 | 0.00 |  | YWHAE | 2.05 | <0.001 |
| POLR2D | 0.36 | <0.001 |  | MAP1LC3B | 2.05 | <0.001 |
| WASH3P | 0.36 | <0.001 |  | TYSND1 | 2.10 | <0.001 |
| ZFAND5 | 0.37 | <0.001 |  | LONP1 | 2.15 | 0.00 |
| BAIAP2 | 0.38 | <0.001 |  | SLC16A1 | 2.16 | <0.001 |
| BRD2 | 0.38 | <0.001 |  | B2M | 2.18 | <0.001 |
| PIK3C2A | 0.39 | <0.001 |  | KDSR | 2.20 | <0.001 |
| PKP1 | 0.39 | <0.001 |  | GARS | 2.20 | <0.001 |
| ZNF593 | 0.40 | <0.001 |  | ARMCX3 | 2.22 | <0.001 |
| PPIL4 | 0.40 | <0.001 |  | ATP5C1 | 2.23 | <0.001 |
| WAC | 0.41 | <0.001 |  | APOC3 | 2.24 | <0.001 |
| EDF1 | 0.41 | <0.001 |  | HAX1 | 2.24 | <0.001 |
| SLAIN1 | 0.41 | <0.001 |  | CALCOCO2 | 2.25 | <0.001 |
| USP36 | 0.42 | <0.001 |  | TIMP1 | 2.25 | <0.001 |
| LTV1 | 0.42 | <0.001 |  | TAX1BP1 | 2.25 | <0.001 |
| CRIP1 | 0.42 | <0.001 |  | MARCKS | 2.25 | <0.001 |
| GINS4 | 0.43 | <0.001 |  | AP2B1 | 2.26 | <0.001 |
| RBM7 | 0.43 | <0.001 |  | LEPRE1 | 2.28 | <0.001 |
| C9orf78 | 0.43 | <0.001 |  | ECM29 | 2.31 | <0.001 |
| PBK | 0.44 | <0.001 |  | SEC61A1 | 2.32 | <0.001 |
| MRPL27 | 0.44 | <0.001 |  | DIAPH1 | 2.35 | <0.001 |
| EIF4ENIF1 | 0.45 | <0.001 |  | NUCB2 | 2.41 | <0.001 |
| WHSC1 | 0.45 | <0.001 |  | RAB11B | 2.47 | <0.001 |
| RPL10 | 0.46 | <0.001 |  | SLC38A2 | 2.57 | <0.001 |
| IRF2BP1 | 0.46 | <0.001 |  | ASS1 | 2.69 | <0.001 |
| RCHY1 | 0.46 | <0.001 |  | GFPT1 | 2.72 | <0.001 |
| PDCL | 0.46 | <0.001 |  | UTS2 | 2.76 | <0.001 |
| ARHGEF1 | 0.46 | <0.001 |  | IARS | 2.82 | <0.001 |

*FDR ≤ 0.01, PEP = posterior error probability
